# Exploring the Effects of the Photochromic Response and Crystallization on the Local Structure of Noncrystalline Niobium Oxide

**DOI:** 10.1021/acsami.4c04038

**Published:** 2024-04-30

**Authors:** Ezgi Onur, Jinsun Lee, Raquel Aymerich-Armengol, Joohyun Lim, Yitao Dai, Harun Tüysüz, Christina Scheu, Claudia Weidenthaler

**Affiliations:** a Max-Planck-Institut für Kohlenforschung, Kaiser-Wilhelm-Platz 1, 45470 Mülheim an der Ruhr, Germany; b Max-Planck-Institut für Eisenforschung, Max-Planck-Straße 1, 40237 Düsseldorf, Germany

**Keywords:** X-ray total scattering, pair distribution function, photochromism, niobium oxide, UV light exposure, amorphous, TT-Nb_2_O_5_, optical band gap

## Abstract

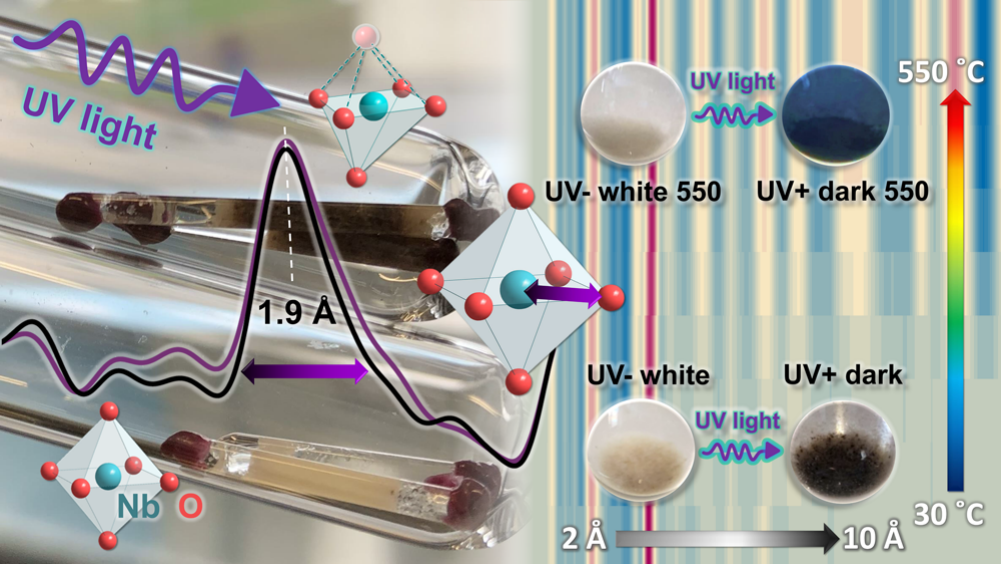

Niobium oxide (Nb_2_O_5_) is a versatile semiconductor material with photochromic properties. This study investigates the local structure of noncrystalline, short-range-ordered niobium oxide synthesized via a sol–gel method. X-ray atomic pair distribution function analysis unravels the structural arrangements within the noncrystalline materials at a local scale. In the following, *in situ* scattering and diffraction experiments elucidate the heat-induced structure transformation of the amorphous material into crystalline TT-Nb_2_O_5_ at 550 °C. In addition, the effect of photocatalytic conditions on the structure of the material was investigated by exposing the short-range-ordered and crystalline materials to ultraviolet light, resulting in a reversible color change from white to dark brown or blue. This photochromic response is due to the reversible elongation of the nearest Nb–O neighbors, as shown by local structure analysis based on *in situ* PDF analyses. Optical band gap calculations based on the ultraviolet–visible spectra collected for both the short-range-ordered and crystalline materials show that the band gap values reduced for the darkened materials return to their initial state after bleaching. Furthermore, electron energy loss spectroscopy reveals the reduction of Nb^5+^ to Nb^4+^ centers as a persistent effect. The study establishes a correlation between the band gap and the structure of niobium oxide, providing insights into the structure-performance relation at the atomic level.

## Introduction

Niobium oxides have various technological applications due to their optical and electrical properties. They have relatively wide band gaps of around 3 eV, high dielectric constants and medium to high refractive indices, making them potential alternatives to titanium oxide photoanodes for dye-sensitized solar cells.^1,2^ They also exhibit a photochromic behavior, i.e., change of color upon exposure to electromagnetic radiation that makes them suitable for applications that require dynamic and reversible optical behavior, such as smart windows, optical data storage, sensors, biomedical applications, and security printing.^3−7^

The origin of photochromism is reported to be related to adsorbed hydrogen atoms that are released from water and organic substances when exposed to light. Moreover, this phenomenon, which has also been observed in other transition metal oxides, is triggered by the photogenerated electron–hole pairs and controlled by the amount of adsorbed hydrogen donor molecules in addition to different oxidation states.^8,9^ The width of the oxide band gap becomes a pivotal factor that profoundly shapes the photochromic process. The determined band gap of niobium oxide films ranges from 2.3 to 4.2 eV in different studies, depending on distinct preparation methods and morphologies.^10−12^

Yao et al. found that niobium oxide thin films exhibit lower background noise than tungsten or molybdenum oxides in chemical sensing devices, despite having a weaker photochromic response.^13^ By using optical waveguides for better detection sensitivity, easier bleaching of niobium oxide in air makes it a suitable candidate material for sensors. Niobium oxides are also promising candidates as electrode materials for lithium insertion in battery research. This is due to their reported ability to provide fast Li-ion transport with better performance characteristics than traditionally used Li-ion battery materials.^14^ Griffith et al. investigated the electrochemical properties of different niobium oxide phases and reported that the tungsten-bronze-like T-Nb_2_O_5_ facilitates high-rate lithium intercalation comparable to nanostructured electrode materials.^15^

**Figure 1:**
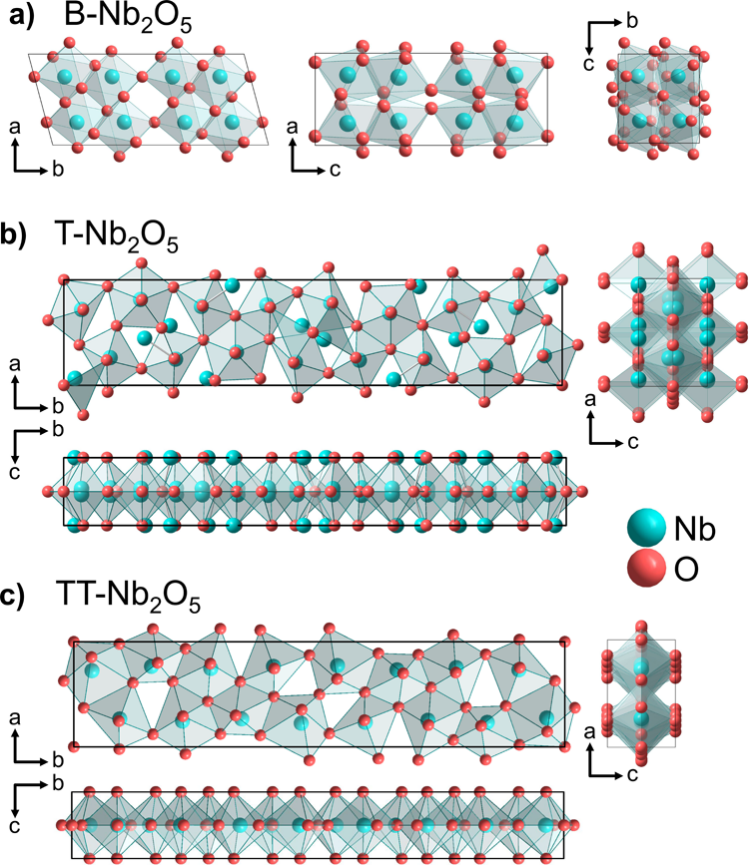
Crystal structure models constructed based on the reported crystallographic data for (a) B-Nb_2_O_5_,^21^ (b) T-Nb_2_O_5_,^23^ and (c) TT-Nb_2_O_5_.^29^

Niobium oxides exhibit diverse crystal structures with distinct wide band gap semiconductor behavior and dielectric properties.^16,17^ The abundance of crystalline phases has led to nomenclature challenges,^5^ with phases categorized by crystal morphologies and German initials based on the temperatures at which these phases appear.^18^ Notably, M-Nb_2_O_5_^18,19^ (with the space group *I*4*/mmm*), B-Nb_2_O_5_^20,21^ (*C*2/*c*) (Figure 1a), and H-Nb_2_O_5_^22^ (*P*2*/m*) are among different crystal structures. H-Nb_2_O_5_ features predominant corner-sharing polyhedra, while the T-Nb_2_O_5_^23^ (*Pbam*) structure (Figure 1b) contains more face-sharing polyhedra. T-Nb_2_O_5_ exhibits a distinctive structure characterized by highly distorted octahedral (NbO_6_) and pentagonal bipyramidal (NbO_7_) niobium environments. It accommodates partially occupied niobium sites together with extended Nb-O interatomic distances. T-Nb_2_O_5_ along with B-Nb_2_O_5_, are recognized as high-pressure modifications of Nb_2_O_5_, with B-Nb_2_O_5_ being stable at low temperatures and T-Nb_2_O_5_ at high temperatures.^20^

Another structure, TT-Nb_2_O_5_ (*Pbam*), is derived by a distortion of the pseudohexagonal modification of Nb_2_O_5_ that is structurally related to columbite.^24^ In another work, TT-Nb_2_O_5_ (Figure 1c) is also reported to be isostructural with δ-Ta_2_O_5_.^25^ The stability of TT-Nb_2_O_5_ depends on impurities like OH^–^, Cl^–^, or vacancies.^26^ The structure comprises Nb-O-Nb chains formed by NbO_6_ or NbO_7_ units along the *c*-axis via corner-sharing polyhedra.^15,27^ Although ongoing research aims to fully comprehend TT-Nb_2_O_5_’s unit cell,^15,28^ it generally represents a less ordered form of T-Nb_2_O_5_,^28^ with detailed discussions on structural insights available elsewhere.^29^

Niobium oxide materials are of great interest also in heterogeneous catalysis as catalysts or as cocatalysts.^30^ In catalysis research, the focus often lies on studying faulted, disordered, or noncrystalline materials. Understanding the structural relationships in catalysis is crucial to linking structure to catalytic performance. The characterization of such materials is challenging due to their lack of long-range order. To overcome this, specialized characterization tools capable of examining fragmented crystal structures are required. X-ray diffraction (XRD) is a widely used technique for analyzing average structures of materials. For noncrystalline materials, X-ray total scattering (TS) data and subsequent pair distribution function (PDF) analysis provide valuable insight into the local structure.

Nanosized or X-ray amorphous niobium oxide also draws attention for its applicability as a photocatalyst.^27,31^ Hydrated niobium oxide, known as niobic acid and used as a precursor material for the synthesis of TT-Nb_2_O_5_, is also extensively investigated as a solid-acid catalyst.^31−33^ Its remarkable catalytic activity is attributed to the formation of surface (OH^–^) groups near distorted Nb-O polyhedra that act as Brønsted acid sites.^32,34^ Aleshina et al. conducted a study on the local structure of short-range-ordered Nb and Ta oxide films using PDF analysis.^35^ They observed similarities between the structural arrangements in amorphous niobium oxide and T-Nb_2_O_5_, both of which exhibited structural units similar to the pseudohexagonal units formed by the metal atoms observed for TT-Nb_2_O_5_. These results suggest the formation of various distorted polyhedra, which in turn leads to the Nb-O chains appearing more curved than in the crystalline material.^11,36^ Furthermore, Llordés et al. explored short-range ordered niobium oxide thin films, demonstrating their potential as flexible electrochromic devices without the need for high processing temperatures.^37^ Their findings revealed a one-dimensional network composed of edge- and corner-sharing NbO_6_ octahedra with terminal Nb=O bonds at the surface. This unique structure was reported to enhance the accessibility of color centers in a one-dimensional network compared to a three-dimensional one. In another study on niobium oxide films, Fernandes et al. demonstrated that induced oxygen vacancies increase film conductivity, leading to more efficient solar cells.^38^

In this study, the local structure of an initially short-range-ordered niobium oxide was investigated together with the changes in the local atomic structure due to exposure to ultraviolet (UV) light stimulating a photochromic response. Additionally, the evolution of the local structure of this material during heat-induced crystallization was investigated by *in situ* temperature-dependent X-ray TS experiments and subsequent PDF analysis. Scanning transmission electron microscopy (STEM) coupled with electron energy loss spectroscopy (EELS) and ultraviolet–visible (UV–vis) spectroscopy were used to gather information on the spatial arrangement and chemical environment of the Nb and O atoms and the light absorption behavior of the niobium oxide. The aim is to gain a comprehensive understanding of structural changes on the atomic scale and their correlation with the changes in band gap due to the photochromic response.

## Results and Discussion

The starting material, niobium oxide (Nb_*x*_O_*y*_) was prepared using niobium (V) ethoxide [Nb(OEt)_5_] as the precursor by a sol–gel route adapted from a previously reported one described in the Experimental Methods.^39^ The as-prepared Nb_*x*_O_*y*_ in suspension is named *UV– white*. The sample codes used for each modification are given in Table 1, which are derived from this sample and counterparts treated under UV light or undergone heat treatment.

**Table 1:** Sample Coding

Sample code	UV light treatment (h)	Air exposure	Heat (°C)	Suspension/Powder
*UV– white*	No	No	No	Suspension
*UV–white P*	No	Yes	60	Powder
*UV+ dark*	2	No	No	Suspension
*UV+ white*	2	Yes	No	Suspension
*UV+white P*	2	Yes	60	Powder
*UV– white550 P*	No	Yes	550	Powder
*UV+ dark 550*	0.5	No	550	Suspension
*UV+ white 550*	0.5	Yes	550	Suspension
*UV+ white550 P*	0.5	Yes	550	Powder

**Figure 2:**
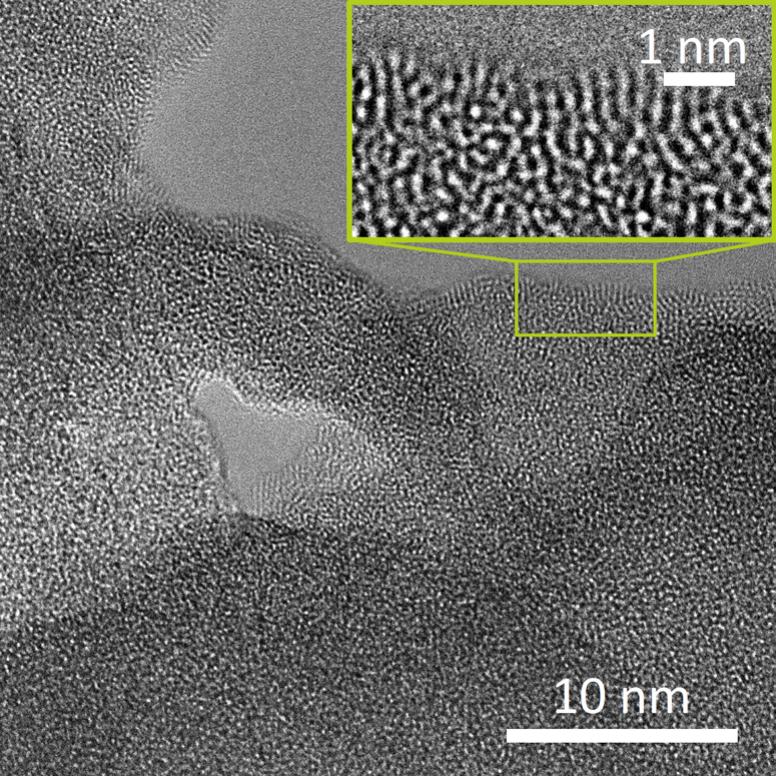
TEM image displaying a region showing short-range ordering in *UV– white P*, higher magnification image is given as an inset.

TEM images of the powder form, *UV– white P*, after separating the solid from the suspension and drying can be seen in Figure 2 displaying the short-range order in the material. A transmission electron microscopy (TEM) image (Figure S3) with lower magnification shows also larger aggregates of 100–200 nm. According to the results of the dynamic light scattering (DLS) (Figure S4) the apparent particle size is 25 nm on average. The data also show the existence of an interconnected porous matrix with diameters around 150 nm, which is consistent with the TEM images. The specific surface area was determined from nitrogen adsorption (for adsorption–desorption isotherms see Figure S5) by using the BET method as 40 m^2^·g^–1^. Assuming spherical isolated particles and a density of ρ = 4.6 g·cm^–3^, this specific surface area corresponds to nanoparticles of 30 nm in diameter, which is comparable to the average particle size of 25 nm obtained from DLS.

**Figure 3:**
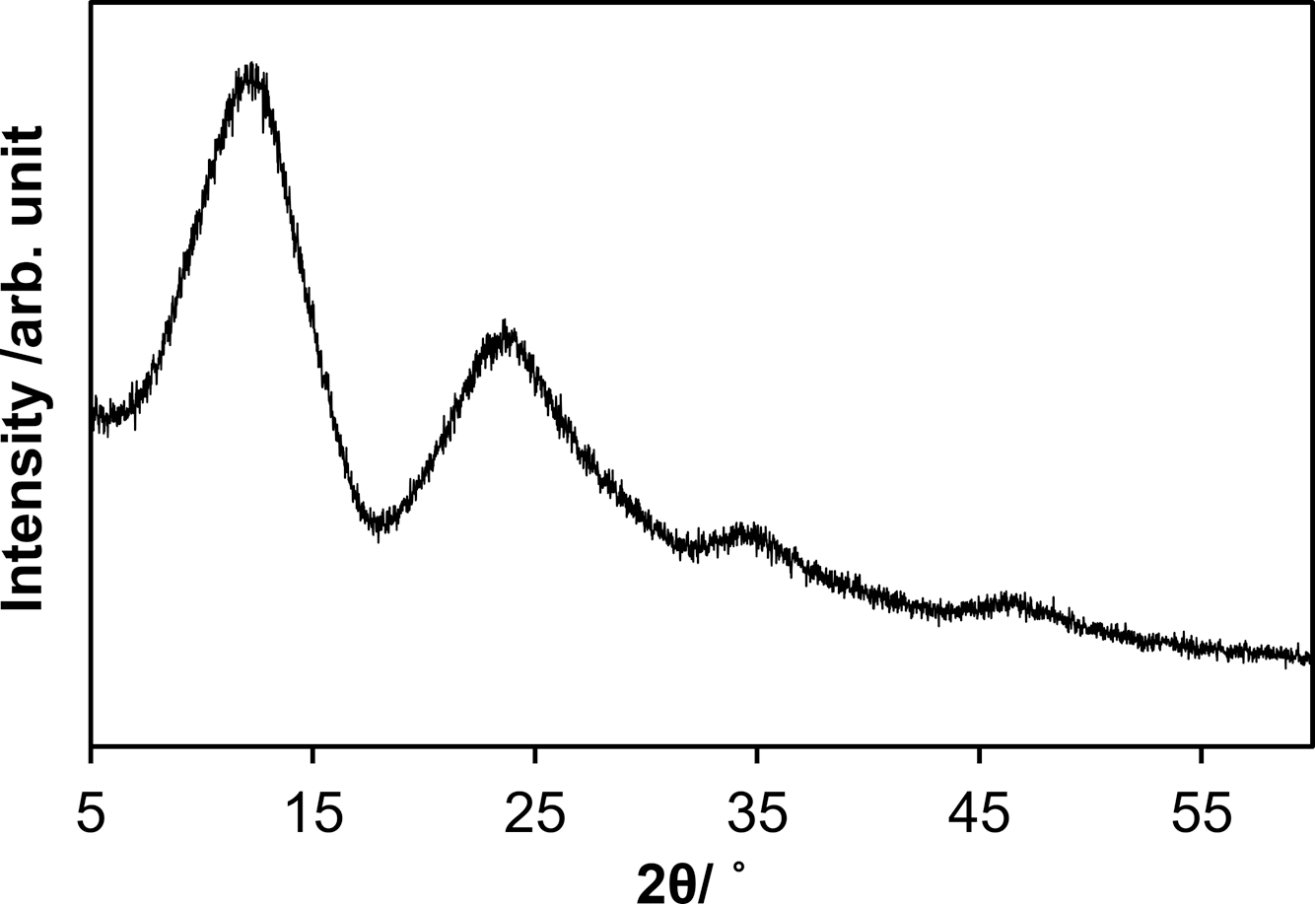
XRD pattern collected from *UV– white P* using an in-house instrument (λ = 0.7093 Å).

The absence of long-range order was confirmed by the powder X-ray diffraction (XRD) pattern (Figure 3, λ = 0.7093 Å) obtained from *UV– white P* lacking Bragg reflections.

**Figure 4:**
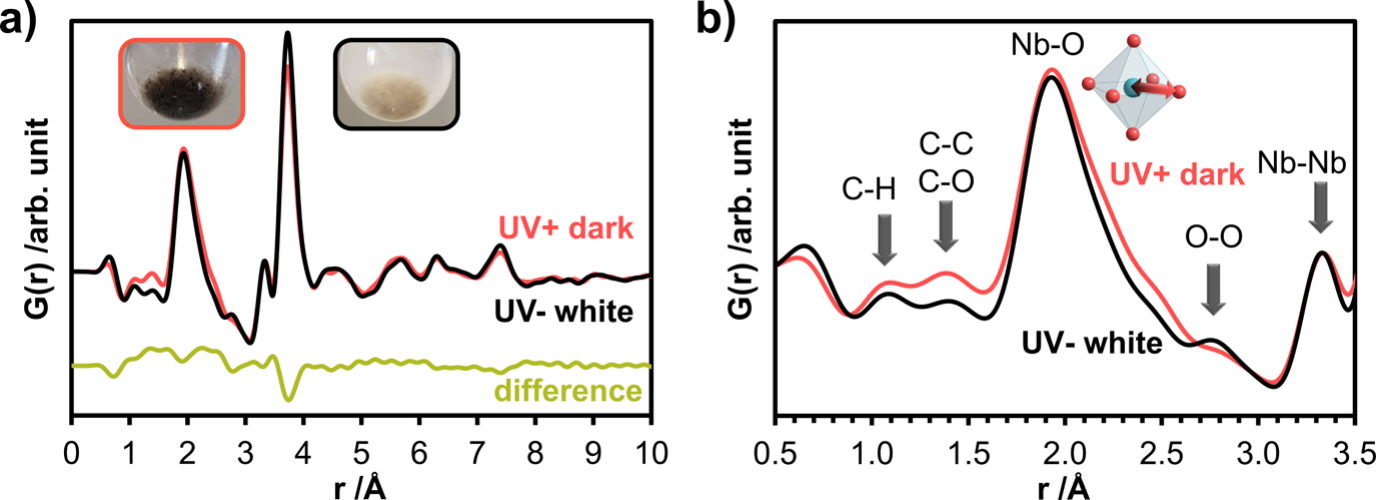
(a) Comparison of the PDFs obtained from *UV– white* (black curve) and *UV+ dark* (coral curve) in reaction suspensions in sealed capillaries. The difference curve obtained by subtracting the PDF intensities is given with an offset. (b) A closer examination of the curve within the 0.5–3.5 Å range, noticeable variations emerge in the intensity of C-containing pair correlations, as well as in the Nb-O and O-O pair correlations.

X-ray total scattering (TS) experiments coupled with PDF analysis provided insight into the short-range ordered and local structural motifs in the noncrystalline state. The PDFs were obtained after processing the data collected from the as-prepared material at a synchrotron facility. The PDF obtained from the as-prepared material, *UV– white*, given in Figure 4a shows pair correlations until 8 Å. The changes observed in the PDFs during the hydrolysis of the liquid niobium ethoxide [Nb(OEt)_5_] precursor are shown in Figure S6. The broad asymmetric peak in the experimental PDF between 1.6 and 2.5 Å corresponds to a distribution of Nb-O distances. This indicates that upon hydrolysis a variety of different polyhedra are formed and/or some polyhedral distortions are introduced. Based on the variety of Nb-O distances reported in literature, noncrystalline Nb_*x*_O_*y*_ likely contains a combination of NbO_6_, NbO_7_ and NbO_8_ units.^35,36^ The Nb-O pair correlations are followed by the region with Nb-Nb pair correlations. Two peaks are observed between 3.0 and 4.2 Å with maxima at 3.3 Å and 3.7 Å (marked with black and yellow arrows in Figure S6). The first corresponds to shorter Nb-Nb pair distances, presumably those in edge-sharing octahedral configuration, while the second can be attributed to Nb-Nb distances between corner-sharing octahedra. Due to the higher intensity observed for the Nb-Nb correlations corresponding to the corner-sharing configuration, this configuration is more frequently observed in the structure. Although a real quantification of the ratio requires a nanometer-scale structure model that would allow PDF baseline subtraction, the ratio of the apparent intensities of the edge- and corner-sharing Nb-Nb pairs gives us an estimated ratio of Nb-Nb_edge_:Nb-Nb_corner_ interactions as 1:3.5. This leads to the possibility of relatively long Nb-O-Nb connections, similar to those discussed for TT-Nb_2_O_5_ layers formed by corner-sharing octahedra, without excluding the existence of edge-sharing Nb-Nb interactions.

A full understanding of this structure can only be achieved through detailed work on cluster modeling and optimization. Although the interatomic distances calculated for a network of connected polyhedra as a cutout of a larger structure model may deviate from those calculated for a cluster in space, simple models can still help to illustrate the possible arrangements.

PDFs calculated from simple cutouts of the B-Nb_2_O_5_ crystal structure show similar pair correlations to those observed in our experimental PDF obtained from *UV– white* (Figure S7). A PDF simulation based on the Nb_12_O_44_H_29_ model constructed by Llordés et al.,^37^ which comprises both edge- and corner-sharing NbO_6_ octahedra, closely matches our experimental PDF. Using density functional theory (DFT)-based molecular dynamics (MD) sampling, the authors investigated various chain-like structures derived from the B-Nb_2_O_5_ crystal structure and finally identified an optimal arrangement in good agreement with the PDF obtained from their amorphous niobium oxide material.^21^ This structure consists of twisted polyhedral blocks arranged in pairs of edge-sharing polyhedra, connected in a zigzag pattern of corner-sharing arrangement. Figure S7 shows a comparison of PDFs simulated for another model, Nb_12_O_50_, representing a cutout of the B-Nb_2_O_5_ crystal structure,^21^ with our experimental PDF from *UV– white*. The model effectively reproduces the observed Nb-O distances and intensity ratios of the different Nb-Nb pair correlations evident in our experimental PDF. However, the PDF based on the model Nb_12_O_44_H_29_ is in better agreement with our data, including the region of the first Nb-Nb pair correlations up to 4 Å.

The intense pair correlations in the experimental PDF up to ∼ 4 Å (Figure 4a) are followed by less intense ones. The latter can be caused by some weak or less frequent interactions within or between domains. To reveal structural details, especially those in the medium-range region, would require modeling of larger structures, which is costly and far beyond the scope of this work.

### Local Structure of Nb_*x*_O_*y*_ and Its Photochromic Response

An evident photochromic phenomenon was observed for *UV– white* when the methanol-containing suspension was irradiated with UV light under inert gas. After an exposure time of several minutes, the color of the suspension changes from white to dark brown (Figure S8). The dark-colored suspension is referred to as *UV+ dark*. The photographs obtained from *UV+ dark* and *UV– white* are shown as insets in Figure 4a. *UV+ dark* loses its dark color when exposed to air if the inert gas purge is not maintained. The sample in this state is named *UV+ white*. Samples of the dark and white suspensions were collected and sealed in capillaries to collect the X-ray TS data at the synchrotron (Figure S9, λ = 0.16167 Å). The data are then processed into PDFs which are given in Figure 4a for *UV+ dark* and *UV– white* (see Figure S10 for a comparison of structure functions and reduced structure functions). The difference PDF curve, which is obtained by the subtraction of PDF intensities of *UV– white* from those of *UV+ dark*, is also given to draw attention to the structural differences observed in combination with the photochromic response.

The PDF obtained for *UV+ dark* shows features up to 8 Å, similar to *UV– white* (Figure 4a). The changes induced by the photochromic response can be understood from the difference curve. The most significant difference is in the Nb-O distances of the first neighbors. Compared to *UV– white*, *UV+ dark* shows a contribution from longer Nb-O distances. The peak corresponding to Nb-O pair correlations is observed between the same values of 1.6–2.5 Å. However, the PDF obtained from *UV+ dark* shows a higher abundance of bonds between 2.0 and 2.5 Å due to the higher intensity of the PDF at these distances. Considering that the Nb-O distances in the reported crystal structures vary between 1.8 and 2.3 Å for NbO_4_, NbO_6_, NbO_7_ and NbO_8_,^22,23^ the structure consists of very long Nb-O distances that make it impossible to accommodate specific Nb and O atoms in the same polyhedron. A closer examination of the range from 0.5 to 3.5 Å shown in Figure 4b reveals that the pair correlations interpreted as C-containing correlations such as C-H and C-O/C-C (centered at 1.1 and 1.4 Å) present a slight increase in intensity in the PDF obtained from the dark suspension. The O-O distances appearing at 2.8 Å in *UV– white* are not observed as strong in the case of *UV+ dark*. This finding is attributed to the disorder that arises in the O-O pair correlations due to the distortion in Nb-O polyhedra. The Nb-Nb correlations, on the other hand, show an overlap at 3.3 Å for the edge-sharing ones but less intense pair correlations at 3.8 Å for *UV+ dark*, corresponding to the corner-sharing octahedra. Similar to the observation of the O-O pairs, this indicates a disorder in the corner-sharing polyhedral chains, possibly related to interlayer interactions induced by UV light. This could be related to the deformation of the polyhedral alignment between the layers caused by the structural changes resulting from the light treatment.

The photochromic behavior of niobium oxide occurs when it is exposed to light, which triggers the formation of hydrogen atoms by dissociation. These hydrogen atoms often originate from hydrogen donor molecules such as methanol adsorbed on the surface of the oxide, which might exist in different stoichiometries. It is noteworthy that the photochromic behavior of niobium oxide synthesized from Nb(OEt)_5_ is particularly unique due to the release of hydrogen atoms from residual organic molecules remaining on the surface after the hydrolysis of the precursor. This behavior is particularly influenced by the electronic band structure, ultimately shaping the resulting photochromic properties.^8,9^

The coloration mechanism of transition metal oxides, when exposed to hydrogen, involves the formation of lower-valence cations. Subsequently, optical transitions occur between adjacent cations with different valence states. In the case of Nb_*x*_O_*y*_, these transitions involve the exchange between Nb^4+^ and Nb^5+^ cations (eq 1):^9^
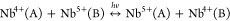
1

The formation of Nb^4+^ species is usually accompanied by some other defects, such as oxygen vacancies in close vicinity of Nb^4+^.^11,40^ The color attained, blue or brown, depends on the crystallinity of the niobium oxide material. The brown color has been observed for the amorphous state, while the blue color is reported for the T- or TT-Nb_*x*_O_*y*_ phase.^41^ Brownish white colors are reported for niobium oxide powders heat treated at temperatures around 400 °C. This phenomenon was assigned to oxygen vacancies generated during the process.^11^ Removal of oxygen atoms from NbO_6_ units, which are considered the basic structural units existing in Nb_2_O_5_ polymorphs, leads to the formation of Nb^4+^ centers with a 5-fold coordination (NbO_5_ polyhedra).^42^ Based on Raman spectroscopy studies performed by Kreissl et al. on various niobium oxides, this can also be explained by structural rigidity associated with the octahedral connection.^42^ Nb-O bond lengths varying between 1.9 and 2.0 Å are reported for structures with little structural strain, consisting mainly of corner-sharing octahedra. On the other hand, a broader distribution of Nb-O bond lengths (1.73–2.26 Å) can be found in more rigid structures, such as H-Nb_2_O_5_, where edge-sharing polyhedra are frequently observed in contrast to *UV+ dark*.^42^ This photochromism phenomenon can also be observed in other transition metal (M) oxides such as WO_3_, MoO_3_ V_2_O_5_, or TiO_2_. Previous work on TiO_2_ prepared similarly by the hydrolysis of a metal ethoxide precursor showed a similar trend in the local structure with the elongated M-O pair correlations upon its photochromic response to UV light.^43^ As the photochromism is triggered by the photogenerated electron–hole pairs, they all show different photochromic responses based on the differences in their electronic structures affecting their reversible coloration-bleaching behaviors.

**Figure 5:**
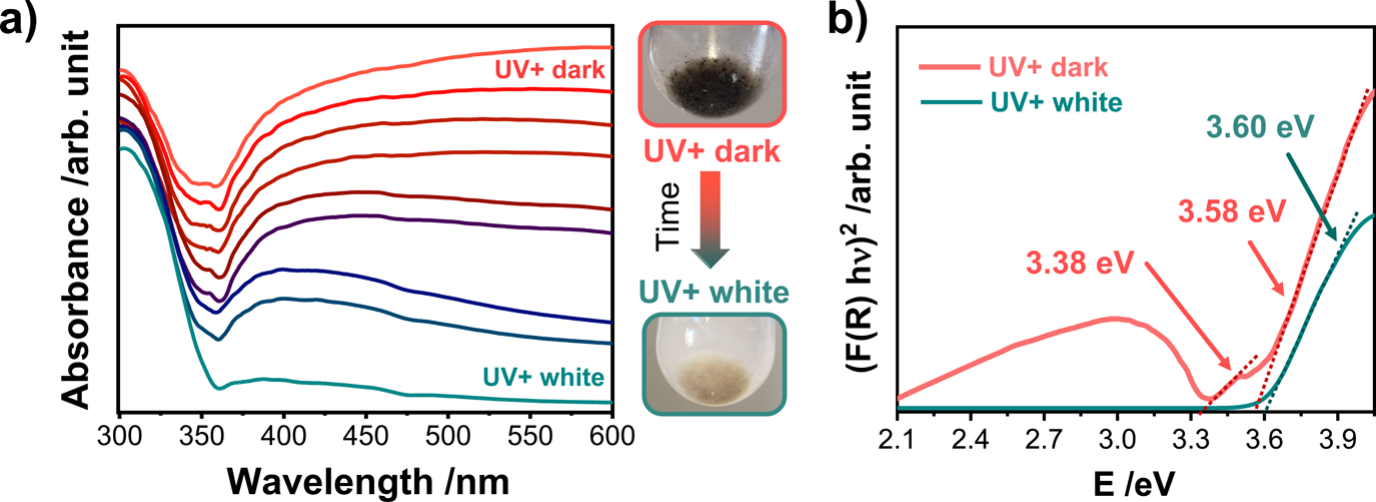
(a) Absorbance spectral profile of *UV+ dark* measured along the 10-min bleaching process to *UV+ white*. (b) Respective (*F*(*R*)*h*ν)^2^ versus energy (*h*ν) plot for determination of the band gap values of *UV+ dark* and *UV+ white*.

Air bleaching is reported to occur by oxidation of niobium hydrogen bronze (H_*z*_Nb_*x*_O_*y*_) which forms in the presence of hydrogen.^9^ It is expected that some surface defects will be induced upon the photochromic response and annihilated by reoxidation. Optical spectroscopy was employed to observe the changes in the electronic band structure of the material during bleaching. The absorbance spectra during the bleaching process of the dark suspension were followed from *UV+ dark* until it became *UV+ white*. The spectral profile acquired during this transformation is provided in Figure 5a. It can be seen that the absorption edge of the spectra changes persistently during bleaching. The excitonic peak observed for *UV+ dark* progressively vanishes as the sample is bleached. The initial and final spectra are processed into the Kubelka–Munk functions (F(R)) and Tauc plots were constructed.^44^ A comparison of the Tauc plots obtained for *UV+ dark* and *UV+ white* (Figure 5b) allows to investigate the differences in excitonic transitions. These deviations in excitonic properties may be attributed to the presence of surface defect states (oxygen vacancies) formed by the reversible photochromic effect and disappear due to the possible annihilation of these defects after bleaching is completed. The optical band gap value for *UV+ dark* derived from the acquired spectra (3.58 eV) and the sub-band gap value (3.38 eV) attributed to the band-to-band transitions from the defect states is found to be smaller than that for *UV+ white* (3.60 eV). These defects introduce additional energy levels between the conduction band (CB) and valence band (VB), thereby influencing the optical properties of the material. Upon exposure to light, electrons are initially excited from the VB to the CB and then, rapidly captured by these traps during the nonradiative relaxation process, resulting in altering the charge carrier dynamics and facilitating the formation of color centers. Furthermore, the existence of intrinsic defects in Nb_2_O_5_ influences the trapping depth and density of color centers, and in turn affects the overall photochromic sensitivity.^7,9^

Similar to the trend observed for the electronic excitations after bleaching, the PDFs obtained from the bleached sample completely overlap with the *UV– white* (Figure S11), indicating that the differences observed in the PDFs due to irradiation change back after bleaching.

Since the photochromic response did not leave any detectable traces in the local structure using the techniques employed thus far, the solid parts of both materials were separated from the suspension for examination with an electron microscope. STEM images obtained from the separated powders are shown in Figure S12. The *UV+ white P* and *UV– white P* samples do not differ in size, shape, and agglomeration.

**Figure 6:**
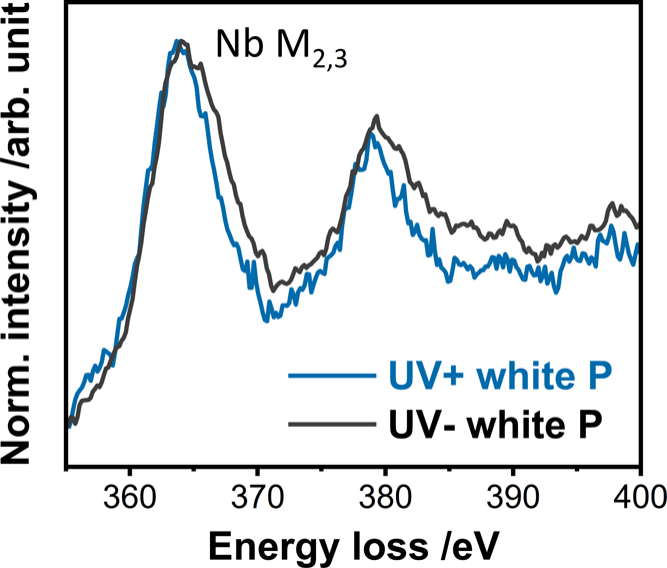
Nb-M_2,3_ edge part of the EEL spectra collected from *UV– white P* and *UV+ white P*.

The band gap calculated for *UV+ white* (3.60 eV) is almost the same as that of the reference sample not treated under UV light, *UV– white P* (3.61 eV) (Figure S13). The TS data (Figure S14) and PDFs (Figure S15) still overlap for the powders, as was the case for the white suspensions (Figure S11). Comparing the EEL spectra collected in STEM mode for both samples revealed no obvious difference in the O-K edge, while the Nb-M_2,3_ edge shown in Figure 6 reveals a peak shift of 1.0 eV to higher energy loss values for *UV– white P* compared to *UV+ white P*. Both spectra show no shoulder at the high energy side of both the M_2_ and M_3_ peaks, in contrast to the spectrum discussed by Bach et al. and Betzler et al. for stoichiometric Nb_2_O_5_ (Nb^5+^). Instead, the broad peaks resemble those found in the EEL spectrum reported in the literature for NbO_2_ (Nb^4+^).^45−47^ The slight shift of the Nb-M_2,3_ lines toward higher energy loss for *UV– white P* compared to *UV+ white P* suggests that the metal centers are less reduced for *UV– white P*. A possible mixture of Nb^4+^ and Nb^5+^ for the *UV– white P* is likely and in the case of Nb^4+^ being the dominant species explaining the missing typical shoulder in the M_2_ and M_3_ peaks. The difference between X-ray TS-PDF and STEM-EELS can be explained by the different length scales. While the collected TS data and the subsequent PDFs correspond to a large volume of the sample under the X-ray beam, EELS data provide more local information collected from individual particles.

### Heat-Induced Crystallization of *UV– White P*

The noncrystalline material *UV– white P* is used as a precursor to obtain information on the evolution of the local ordering during crystallization. *In situ* temperature-dependent in-house XRD experiments show the first reflections of crystalline phases at 540 °C upon heating (Figure S16a, λ = 1.54186 Å). After cooling from 550 °C to room temperature, the obtained sample was called *UV– white 550 P*.

The temperature at which the first reflections are observed aligns with the results of the thermal analysis performed on *UV– white P*, which indicated the onset of crystallization between 530 and 560 °C, varying depending on the heating rates (Figure S17a). A single-step weight loss of 10–15 wt% was measured up to 450 °C. Based on the comparison of the FTIR spectra collected from *UV– white P* and *UV– white 550 P* (Figure S18), it can be inferred that this weight loss can be attributed to the removal of water. Based on the crystallization peaks obtained at different heating rates, an apparent activation energy of E_a_ = 347 kJ·mol^–1^ was calculated^48^ (Figure S17b) similar to the values reported in the literature.^49^

Rietveld refinements were performed on the diffraction data of *UV– white 550 P*, testing T-Nb_2_O_5_^23^ and TT-Nb_2_O_5_^29^ as structure models (Figure S16b and Figure S16c). The results of the Rietveld refinements (refer to Table S1 for refined parameters) showed that the real structure exhibits deviations from these two model structures. However, the refinement of the lattice parameters to values similar to the model structures indicates that the real structure is orthorhombic, and for simplification, the TT-Nb_2_O_5_ structure was used for further analysis.

**Figure 7:**
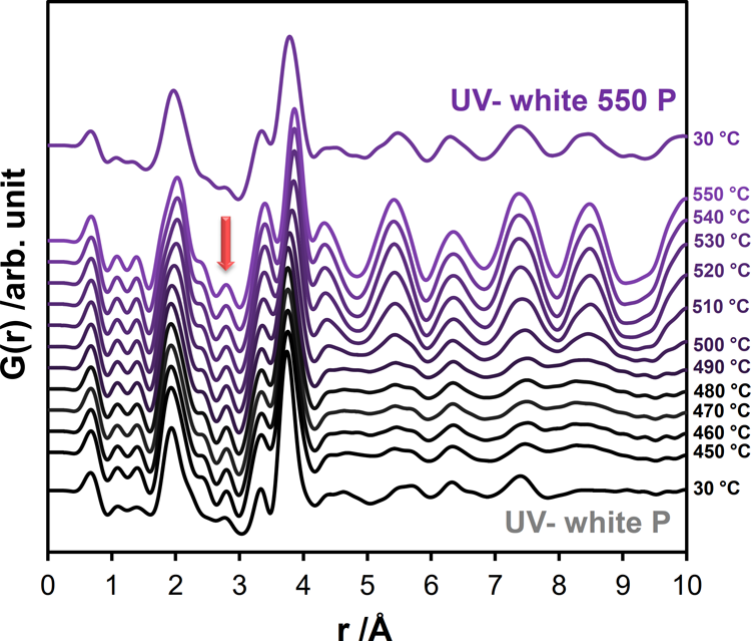
PDFs calculated from the scattering data (Figure S19a) obtained from *in situ* heating experiments on *UV– white P* during the transformation into *UV– white 550 P*. The experiments were conducted with a starting temperature of 30 °C, with temperature steps of 10 °C between 50 and 450 °C, and temperature steps of 50 °C between 450 and 550 °C. The arrow shows O-O pair correlations at about 2.8 Å.

*In situ* temperature-dependent X-ray TS experiments were performed on *UV– white P* to analyze the evolution of the local structure during crystallization and to obtain the heat-treated material, *UV– white 550 P*. The collected TS data (Figure S19a, λ = 0.20723 Å) were processed into PDFs, which are shown in Figure 7. When examining the series of PDFs, it becomes evident that the pair correlations observed at room temperature up to a distance of 4 Å remain largely intact during the initial heating stages, showing only the effects of thermal motion. Around 490 °C, subtle features above 4 Å begin to increase in intensity. Above 500 °C, these features become visible, along with the change in the profile of the first Nb-O pair correlations (Figure S19b).

As the temperature rises, the intensity of the O-O pair correlations at about 2.8 Å gradually increase and finally form a distinct peak at 450 °C, indicating the ordering of oxygen atoms. Following the O-O pair correlations, two peaks with maxima at 3.3 Å and 3.7 Å corresponding to the edge- and corner-sharing octahedral configurations remain at similar distance values. However, the comparison of the intensities of the two peaks indicates that the prevalence of the edge-sharing configuration increases, while the corner-sharing octahedral connection becomes less frequent. This transition could indicate a decrease in the number of connections along the *c*-axis of the TT-Nb_2_O_5_ unit cell, giving the structure greater flexibility. This shift toward a higher frequency of edge-sharing octahedra improves the overall stability of the structure and is consistent with the increased flexibility associated with the corner-sharing octahedral configuration as reported in the literature.^50^

**Figure 8:**
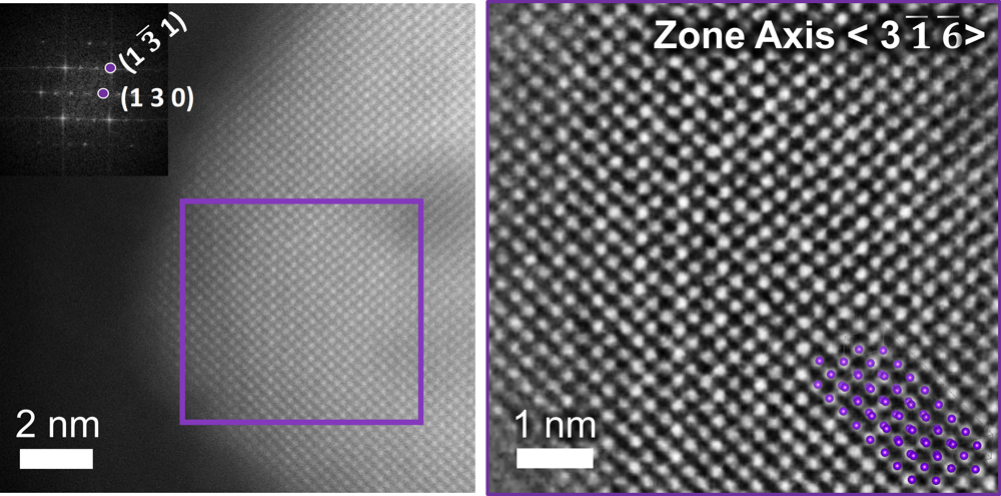
HAADF-STEM images of a crystalline region of *UV– white 550 P* corresponding to TT-Nb_2_O_5_ viewed in ⟨3 −1 −6⟩ zone axis.

An increase in the size of the crystalline domains as the temperature increases is evident in the long-range PDFs (Figure S19b). Moreover, no pair correlations suggesting the formation of intermediate crystalline structures are observed during the transformation into TT-Nb_2_O_5_. To elucidate the spatial arrangement of these ordered regions, *UV– white 550 P* was examined with high-resolution high-angle annular dark field (HAADF) STEM. The study revealed the presence of ordered regions exhibiting the crystal structure corresponding to TT-Nb_2_O_5_. The fast Fourier transformation (FFT) of the STEM image revealed a good agreement in terms of interplanar spacing and angles of TT-Nb_2_O_5_ viewed in ⟨316⟩ zone axis (Figure 8).

**Figure 9:**
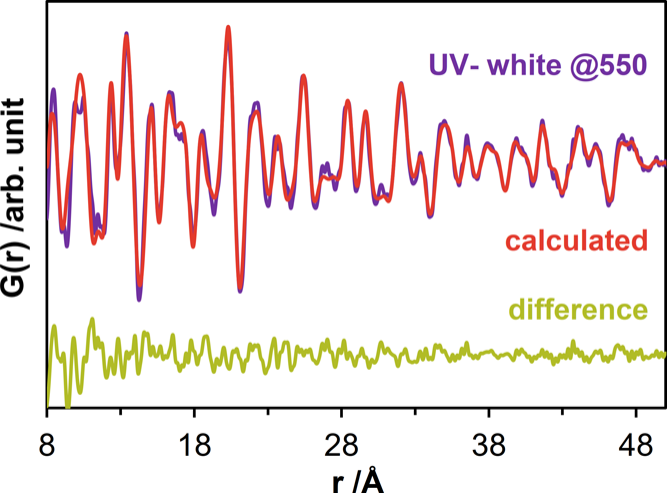
Refinement of the PDFs obtained from *UV– white 550 P* (the sample at 30 °C after heating during the *in situ* temperature-dependent total scattering experiments) using TT-Nb_2_O_5_^29^ crystalline model structure for the 8–50 Å range (*R*_w_ = 0.24). Calculated and difference curves are displayed in red and green.

Based on this information, the TT-Nb_2_O_5_ structure model was refined against the experimental PDF obtained for *UV– white 550 P* (see Figure S20 for total PDF simulations). The refinement was first restricted to the range between 8 and 50 Å to exclude possible contributions from the noncrystalline pair correlations. The fit in Figure 9 shows a reasonable match (*R*_w_ = 0.24) (refined parameters given in Table S2). Later, the refined values were used to fit the data within the range between 0.5 and 20 Å, (except for the scale factors) to estimate the contribution of the crystalline part to the short-range pair correlations. The fit of the short-range data (Figure S21) shows a higher mismatch (*R*_w_ = 0.74). An examination of the difference curve shows similarity to the PDF obtained from *UV– white P*. This indicates that even after the heat treatment, the material still contains a significant contribution from the short-range ordered regions.

### Heat-Treated Nb_2_O_5_ and Its Photochromic Response

**Figure 10:**
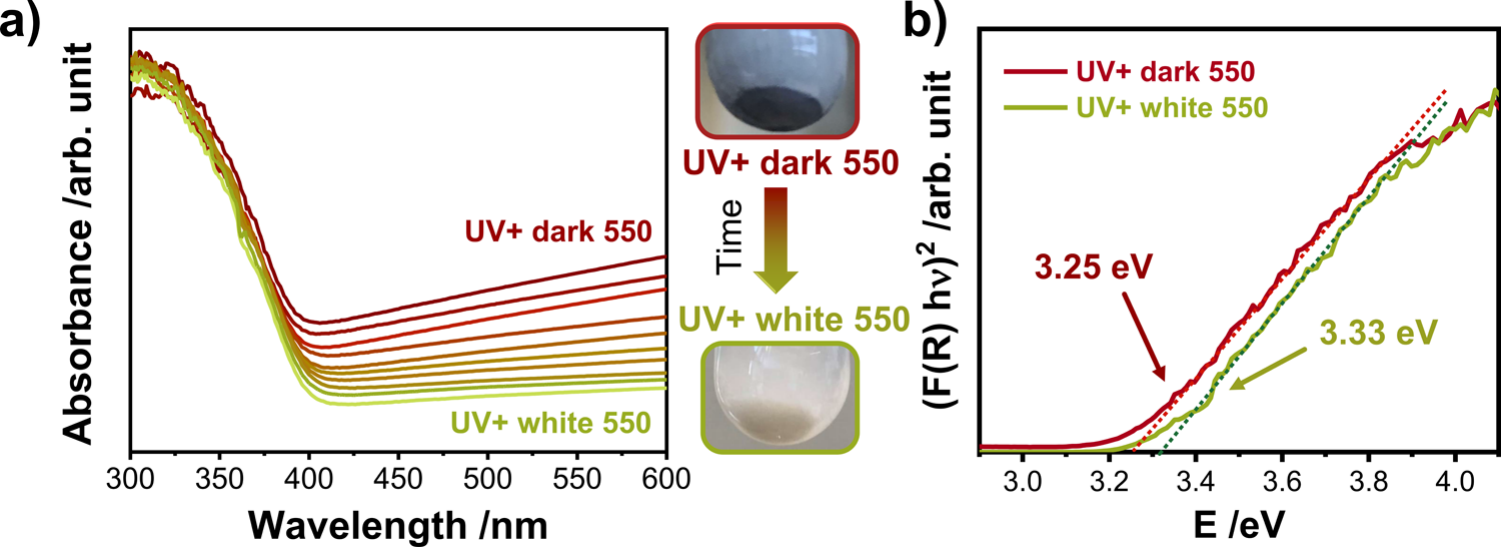
(a) Absorbance spectra of *UV+ dark 550* measured along the 60-min bleaching process to *UV+ white 550*. (b) Respective Tauc plots to determine the band gap values of *UV+ dark 550* and *UV+ white 550*.

Apart from the changes in the local structure, it is also important to obtain information on how the photochromic behavior changed after crystallization. The suspension containing the heat-treated powder *UV– white* 550 was exposed to UV light, resulting in a dark-colored sample named *UV+ dark 550*. It was observed that the sample turned more bluish rather than brownish, consistent with expectations reported elsewhere.^41^ However, the dark blue suspension in this state is also sensitive to air and can only be preserved under inert gas purging. For comparison with the noncrystalline counterpart, spectral absorbance profiles were also collected during the bleaching of *UV+ dark 550* in air to obtain *UV+ white 550* (Figure 10a). The value of the band gap calculated from the Tauc plots is shown in Figure 10b. The first observation is the absence of an excitonic peak in the spectra of *UV+ dark 550*, as observed for *UV+ dark*. Furthermore, the band gap value for the dark suspension, 3.25 eV, was found to be smaller than that calculated for the noncrystalline dark material (3.38 eV). This can be explained by the annihilation of the oxygen vacancies at elevated temperatures during the growth of the crystalline domains under the air supply.

The photochromic effect is also reversible for the heat-treated material. The calculated band gaps for the powders, *UV+ white 550 P* and *UV– white 550 P*, give the same values of 3.36 eV (Figure S22). Similarly, the comparison of the XRD patterns does not show a noticeable difference (Figure S23).

The formation of a distorted TT-Nb_2_O_5_ phase during heat-induced crystallization of *UV– white P* demonstrates the tunability of the phase composition for potential applications. The distorted structure shows a variety of possible linkages of the polyhedra (Figure 1a and Figure 1b), leading to variations in polyhedral connections and the presence of defects, ultimately influencing the band gap and absorption spectra. Mechanisms involving the formation of hydrogen-related defects, changes in oxidation states, and their role in coloration and bleaching processes are examined, underscoring the reversibility of the photochromic behavior, which is particularly promising for applications requiring dynamic optical behavior.

Moreover, *UV– white P* demonstrates thermal stability up to 500 °C. The ability to maintain the desired phase composition at elevated temperatures is considered a positive feature that offers greater flexibility in materials engineering. Overall, understanding the structure at such a small scale is essential for optimizing these materials for various applications.

## Conclusion

In this study, we have investigated the structural and band gap changes induced by the photochromic response in noncrystalline niobium oxide upon exposure to UV light and heat-induced crystallization. Atomic pair distribution functions derived from X-ray TS experiments of the amorphous materials reveal intense pair correlations in the short-range-ordered solid, particularly within interatomic distances of 4 Å demonstrating the presence of a variety of polyhedral motifs, including NbO_6_, NbO_7_, and NbO_8_ units.

The short-range-ordered, X-ray-amorphous niobium oxide exhibits thermal stability up to 500 °C, making it an attractive candidate for applications requiring performance at higher temperatures. *In situ* temperature-dependent TS and XRD experiments illustrate the crystallization of short-range-ordered material into the TT-Nb_2_O_5_ structure. Nevertheless, even after reaching a temperature of 550 °C, a considerable amount of the short-range-ordered solid persists. The change in the crystallinity of the material results in alterations in the band gap and absorption spectrum of the material.

During UV illumination, both noncrystalline and heat-treated niobium oxide suspensions exhibit a reversible color change from white to blue or brown, reflecting the photochromic response. By conducting PDF analysis of the X-ray-amorphous starting material, we were able to elucidate the reversible expansion of the first Nb-O bond distances responsible for the coloration. The UV–vis spectroscopy experiments performed under bleaching conditions facilitated the correlation between the band gap with the structural state. The band gaps obtained for the dark suspensions are smaller than those observed in the bleached samples, with the band gaps being even smaller in the heat-treated material. This reversibility, which is associated with changes in band gap, shows potential for applications that demand dynamic optical behavior.

Our results have significant implications for understanding changes in the electronic and optical properties of materials with photochromic behavior. This research is expected to lay the foundation for future investigations on structural adaptations and material properties and provide valuable insights into the photochromism of niobium oxides. We anticipate that our study will contribute to revealing the mechanisms that govern the optical properties and crystallization behavior, facilitating new applications in optoelectronics and related fields and promising future advances.

## Experimental Methods

### Preparation Procedure

The synthesis method which was explained elsewhere,^39^ so-called ‘direct injection’, is a simple synthesis procedure to obtain high surface area metal oxide photocatalysts involving simultaneous tests for photocatalytic water splitting. The method which is adapted to the Nb-O system, involves direct injection of niobium(V) ethoxide [Nb(OEt)_5_] precursor into the water–methanol reaction mixture in a photocatalytic reaction cell. The reaction cell is equipped with a lamp that radiates ultraviolet (UV) light. The direct injection method involves a photocatalytic test where the catalyst is exposed to UV light for 2 h. Therefore, during the synthesis, the catalyst suspension is illuminated under UV light for 2 h. This suspension is referred to as “*UV+ dark*”. As a non-UV counterpart, samples were also synthesized in the absence of UV light which is referred to as “*UV– white*”. Both synthesis routes are explained below in detail. The samples resulting from the two synthesis routes were collected for stationary suspension measurements in sealed capillaries. A protective atmosphere was maintained in the case of air-sensitive (dark-colored suspension subjected to UV irradiation) samples. The sediments of the suspensions were separated by decanting the liquid part and were dried overnight (for 3 h in case of powders obtained as counterparts to suspensions measured at the synchrotron source due to limited time) at 60 °C in the oven to obtain “*UV+ white P*” and “*UV– white P*” powder samples.

### Procedure under UV Light Exposure (*UV+ Dark*)

Synthesis of samples involves injection of 380 μL Nb(OEt)_5_ (99.95%, Sigma Aldrich, CAS: 3236–82–6) as metal oxide precursor into a mixture of 180 mL deionized water and 20 mL MeOH (Sigma Aldrich, CAS: 67–56–1) in a glass photocatalytic reaction vessel. This concentration was calculated aiming at 1 g·L^–1^ Nb_2_O_5_ catalyst in the end. The reaction vessel is equipped with a Peschl Ultraviolet TQ150 150 W middle-pressure Hg-lamp having peak emission at 366 nm (240–577 nm). The UV lamp is inserted into the reaction vessel with a water-cooled jacket. Following the injection of the precursor into the reaction solution, the UV lamp is turned on. The suspension was exposed to UV light for 2 h. During illumination, reaction suspension was stirred using a stirring bar and kept under continuous Ar purging (50 mL·min^–1^). This sample attains a dark color (brown) and when Ar purging is not maintained, changes color back to white and is named as “*UV+ white*”.

### Procedure without UV Light Exposure (*UV– White*)

To observe the effects of UV light exposure, the synthesis was also carried out in the absence of UV light. The same amount of the precursor was injected into the reaction mixture prepared in the same way. In this case, samples were synthesized in a beaker rather than the reaction vessel. The reaction mixture was stirred for 2 h under continuous Ar purging. In this way, “*UV– white*” samples were obtained as a counterpart and used for further local structure analysis. These samples together with the dried version, “*UV– white*”, are studied extensively including the *in situ* heating experiments in the present work.

### Experimental Parameters for the Total Scattering Experiments

#### Room Temperature Total Scattering Data and PDFs

Data collection for the powder samples, suspensions and liquid precursor was performed at the I15–1 beamline at Diamond Light Source (Diamond). The energy was 77 keV (λ = 0.16167 Å), the sample to detector distance (SDD) was 200 mm (Q_min_ = 0.2 Å^–1^, Q_max, instrumental_ = 38 Å^–1^, Q_max,_ = 22 Å^–1^, Q_damp_ = 0.0258 Å^–1^, Q_broad_ = 0.0118 Å^–1^), and the beam size on the sample was 700 × 150 μm. Data acquisition was performed with a Perkin-Elmer XRD 4343 CT detector. Collected scattering data were integrated using the DAWN software package.^51^ Scattering data obtained from empty capillary and capillary filled with a water–methanol mixture were used as backgrounds for powder and suspension measurements, respectively. The program PDFgetX3^52^ implemented in the xPDFsuite^53^ was used for processing PDFs from the integrated scattering data. Diffpy-CMI^54^ was used to simulate the PDFs from the cluster models using Q_max,_ = 22 Å^–1^, Q_damp_ = 0.0258 Å^–1^, Q_broad_ = 0.0118 Å^–1^.

The synchrotron data for *UV– white* 500 P (sample cooled down after the *in situ* heating experiments) were collected *ex-situ* in a sealed 0.5 mm borosilicate glass capillary at beamline P02.1 at PETRA III, Deutsches Elektronen-Synchrotron (DESY) [energy: 60 keV (0.2071 Å); detector: Varex XRD 4343 CT; SDD: 252.62 mm; Q_min_ = 0.65 Å^–1^, Q_max, instrumental_ = 33.65 Å^–1^, Q_max,_ = 22 Å^–1^, Q_damp_ = 0.0304 Å^–1^, Q_broad_ = 0.00253 Å^–1^, beam size: 1 × 1 mm]. The DAWN software package was used to integrate scattering data.

#### In Situ Temperature-Dependent Total Scattering Data and PDFs

*In situ* heating experiments were performed on “*UV– white*” at beamline P02.1 at PETRA III, DESY [energy: 60 keV (λ = 0.20723 Å); detector: Perkin-Elmer XRD 1621; SDD: 250.88 mm; Q_min_ = 0.4 Å^–1^ and Q_max, instrumental_ = 34.2 Å^–1^, beam size: 1 × 1 mm]. Powder samples were packed in quartz capillaries (both ends open, length 100 mm, inner diameter 0.9 mm, wall thickness 0.15 mm) ensuring a few centimeters of sample length. The capillaries are mounted on the custom-made cell and are heated at a rate of 10 K·min^–1^ using a hot air blower. Temperature was calibrated based on the trend obtained from the reading of the thermocouple inserted inside the empty quartz capillary heated in the same way as the samples. The temperature calibration curve and temperature vs time curve are given in Figure S1 and Figure S2.

During heating, the powder was kept under synthetic air with a flow of 5 mL·min^–1^. The sample obtained after cooling down to the room temperature is named as “*UV– white 500 P*”. Data were collected in temperature steps of 10 °C between 300 and 400 °C. The data collection time per frame was 5 min. The DAWN software package was used to integrate scattering data. PDFs were generated (q_max_ = 22 Å^–1^) using PDFgetX3 implemented in the xPDFsuite, simulated (q_max_ = 22 Å^–1^, ADPs= 0.003, delta2 = 1.0, Q_damp_= 0.0375 Å^–1^, Q_broad_= 4.067e-06 Å^–1^) and refined using PDFgui^55^ implemented in the xPDFsuite.^53^

#### In Situ XRD Experiments

*In situ* temperature-dependent XRD experiments were performed using Anton Paar XRK 900 reaction environment attached to a Rigaku SmartLab diffractometer equipped with a rotating anode (9 kW, 45 kV, 200 mA) operated in the Bragg–Brentano geometry (Cu Kα_1,2_, λ = 1.54186 Å) with a Cu Kβ filter. The powder sample was placed on a MACOR sample holder 6 mm in diameter, which was heated from 30 to 550 °C at a 10 °C·min^–1^ heating rate under a continuous flow of synthetic air at a rate of 10 mL·min^–1^. Data were collected at each 50 °C between 50–450 °C, at each 10 °C between 450–550 °C and at 30 °C before and after the heating experiment in the 20–60° 2θ range at a scan rate of 5°·min^–1^ (step size, 0.01°) with a HyPix-3000 multidimensional detector (one-dimensional mode). The Rietveld refinements were performed using DiffracPlus TOPAS 6 software (Bruker AXS GmbH, Karlsruhe, Germany).^56^

### UV–Vis Spectroscopy and Calculating the Optical Band Gap

*UV– white* and *UV+ dark* samples were prepared and small parts of the suspensions with higher powder content were transferred onto the glass holders under Ar flow. The suspension sandwiched in between two quartz glass sample holders was then mounted on the spectrometer in a glovebox. The bleaching process lasted for 10 min, during which spectra were collected. *UV– white 500 P* sample was redispersed in the water–methanol mixture and was subjected to UV light for 0.5 h. During illumination, reaction suspension was stirred using a stirring bar and kept under continuous Ar purging. This sample attains a dark color (blue) and when Ar purging is not maintained, changes color back to white and is named as “*UV+ white 550*”. Small parts of the two suspensions with higher powder content were transferred onto the glass holders under Ar flow. The suspension sandwiched in between two quartz glass sample holders was then mounted on the spectrometer in a glovebox. The bleaching process lasted for 60 min, during which spectra were collected.

The data acquisition procedure was repeated for the powders obtained from the respective samples.

The UV-DRS profiles were acquired at room temperature, spinning a wavelength range of 300–800 nm, utilizing a PerkinElmer Lambda 365 UV–vis spectrophotometer. BaSO_4_ (spectroscopy grade) served as the reference material. The Kubelka–Munk theory guided the construction of Tauc plots for a direct semiconductor, where [F(R)·hν)]^2^ was plotted against hν (incident photon energy). Here, F(R) is defined as (1 - R)^2^ /2R), with R representing the measured reflectance.^44^

### Transmission Electron Microscopy Examination

High-resolution TEM imaging on *UV– white P*, *UV+ white P* and *UV– white 550 P* was done using a Titan Themis 80–300 instrument (Thermo Fisher Scientific) operated at 300 kV and equipped with a Cs corrector for the image-forming lens. HAADF images and EELS data were taken in a probe-corrected Titan Themis 80–300 instrument (Thermo Fisher Scientific) at an acceleration voltage of 300 kV. The EELS data were acquired with a Gatan Quantum ERS energy filter with an entrance aperture collecting electrons up to 35 mrad and a dispersion of 0.25 eV per channel.

### Nitrogen Sorption Experiments

The specific surface area was determined by nitrogen sorption experiments with a Quantachrome NOVA 3200e instrument after degassing approximately 160 mg powder at 150 °C overnight. Data were evaluated by the BET (Brunauer–Emmett–Teller) method using the adsorption data in the relative pressure range of 0.05 to 0.2. From the specific surface area approximate particle sizes can be calculated using the relation D_p_= 6000·σ^–1^·A_s_^–1^ (assuming spherical particles, D_p_ = particle size in nm, σ = specific density in g·cm^–3^, A_s_ = specific surface area m^2^·g^–1^).

### Dynamic Light Scattering Experiments

Dynamic light scattering (DLS) data of *UV– white* were recorded on a Malvern Zetasizer Nano-ZS using laser radiation with a wavelength of 633 nm. The scattered light was measured at a backscattering angle of 173°. The suspension collected after synthesis was ultrasonicated for 30 min.

### Thermal Analysis

Thermogravimetry (TG) and differential scanning calorimetry (DSC) measurements of *UV– white P* were carried out using a NETZSCH STA 449 F3 Jupiter thermal analyzer for the qualitative analysis of crystallization temperatures. The measurements were carried out under an airflow of 40 mL·min^–1^ using approximately 10 mg powder heated in an aluminum oxide crucible with heating rates of 2, 5, and 10 °C·min^–1^.

### FTIR Spectroscopy

Fourier transform infrared (FTIR) spectroscopy measurements of *UV– white P* and *UV– white 550 P* were performed using a Perkin-Elmer Spectrum Two spectrometer with an attenuated total reflectance (ATR) unit.

### Structure Model Visualization and Modification

Diamond Crystal and Molecular Structure Visualization software^57^ is used for the visualization and modification of the structure models.

## Supporting Information Available

Figure S1: Temperature calibration curve used for setting the temperature to adjust the set temperatures for the hot air blower; Figure S2: Temperature vs time curve for the 30–550 °C *in situ* temperature-dependent experiments; Figure S3: TEM image obtained from *UV– white P* displaying the interconnected porous matrix; Figure S4: Particle size distribution based on the results of the dynamic light scattering obtained from *UV– white* after ultrasonication.; Figure S5: Nitrogen adsorption/desorption isotherms of *UV– white P* sample; Figure S6: Experimental PDFs obtained from the liquid Nb(OEt)_5_ precursor and the *UV– white* sample; Figure S7: PDFs simulated for (a) Nb_12_O_50_, a cutout from the B-Nb_2_O_5_ structure and (b) Nb_12_O_44_H_29_ cluster model constructed by Llordés et al.^10^ in compared to the experimental PDF obtained from *UV– white*; Figure S8: Photographs taken during the preparation of *UV+ dark*; Figure S9: Comparison of the X-ray TS curves obtained from *UV– white*, *UV+ dark* and *UV+ white*; Figure S10: Comparison of the (a) total scattering structure functions (S(Q)) and (b) the reduced structure functions (F(Q)) obtained from *UV– white* (black curve) and *UV+ dark* (coral curve) during processing the total scattering data into PDFs; Figure S11: Comparison of the PDFs obtained from *UV– white* and *UV+ white*; Figure S12: Comparison of morphologies of *UV– white P* and *UV+ white P* examined by HAADF STEM; Figure S13: Tauc plots to determine the band gap values of *UV+ white P* and *UV– white P*; Figure S14: X-ray TS data obtained from *UV+ white P* and *UV– white P*; Figure S15: PDFs obtained from *UV+ white P* and *UV– white P*; Figure S16: Diffraction patterns collected during *in situ* temperature-dependent X-ray diffraction experiments performed on *UV– white P* to obtain *UV– white 550 P* and the Rietveld refinement results; Table S1: Parameters used for the refinements of the XRD pattern obtained from *UV– white 550 P*; Figure S17: DSC and TG data collected for *UV– white P*; Figure S18: FTIR spectra obtained using ATR mode from *UV– white* 500 P and *UV– white P*; Figure S19: Experimental X-ray total scattering data and the respective long-range PDFs obtained from *in situ* temperature-dependent experiments performed on *UV– white P*; Figure S20: PDFs simulated for T-Nb_2_O_5_ and TT-Nb_2_O_5_ crystal structure models; Table S2: Parameters used for the refinements of the PDF obtained from *UV– white 550 P*; Figure S21: Refinement of the PDFs obtained from *UV– white 550 P*; Figure S22: Tauc plots to determine the band gap values of *UV+ white 550 P* and *UV– white 550 P*; Figure S23: Comparison of the XRD patterns collected from the *UV– white 550 P* and *UV+ white 550 P* (PDF)

## Abbreviations

Nb_2_O_5_: niobium pentoxide
Nb_*x*_O_*y*_: niobium oxide
XRD: X-ray diffraction
TS: total scattering
PDF: pair distribution function
UV: ultraviolet
STEM: scanning transmission electron microscopy
EELS: electron energy loss spectroscopy
UV–vis: ultraviolet–visible
TEM: transmission electron microscopy
DLS: dynamic light scattering
M: metal
DFT: density functional theory
MD: molecular dynamics
HAADF: high angle annular dark field
FFT: fast Fourier transformation.
