# Supplementary material for: Exploring the Effects of the Photochromic Response and Crystallization on the Local Structure of Noncrystalline Niobium Oxide
Source: ACS Appl Mater Interfaces. 2024 Apr 30;16(19):25136–47. doi: 10.1021/acsami.4c04038 (PMC11103654; doi:10.1021/acsami.4c04038)
Supplement: Supplementary file 1 — am4c04038_si_001.pdf [file am4c04038_si_001.pdf]

Supporting Information

Exploring the effects of the photochromic response and crystallization on the local structure of non-crystalline niobium oxide

Ezgi Onur,[a] Jinsun Lee,[a] Raquel Aymerich-Armengol,[b] Joohyun Lim,[b,c] Yitao Dai,[a,d] Harun Tüysüz,[a] Christina Scheu[b] and Claudia Weidenthaler[*a]

Affiliations
[a]Max-Planck-Institut für Kohlenforschung, Kaiser-Wilhelm-Platz 1, 45470 Mülheim an der Ruhr, Germany

[b]Max-Planck-Institut für Eisenforschung, Max-Planck-Straße 1, 40237 Düsseldorf, Germany

[c]Department of Chemistry, Institute for Molecular Science and Fusion Technology, Multidimensional Genomics Research Center, Kangwon National University, Chuncheon 24341, Republic of Korea

[d]Suzhou Institute for Advanced Research, University of Science and Technology of China, Suzhou, Jiangsu 215123, People's Republic of China

*Corresponding author: weidenthaler@mpi-muelheim.mpg.de

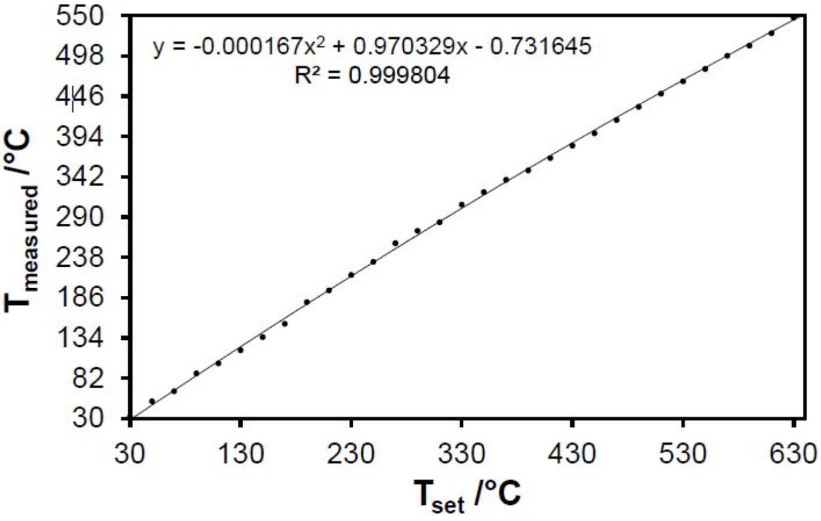

Figure S1 Temperature calibration curve used for setting the temperature to adjust the set temperatures for the hot air blower.

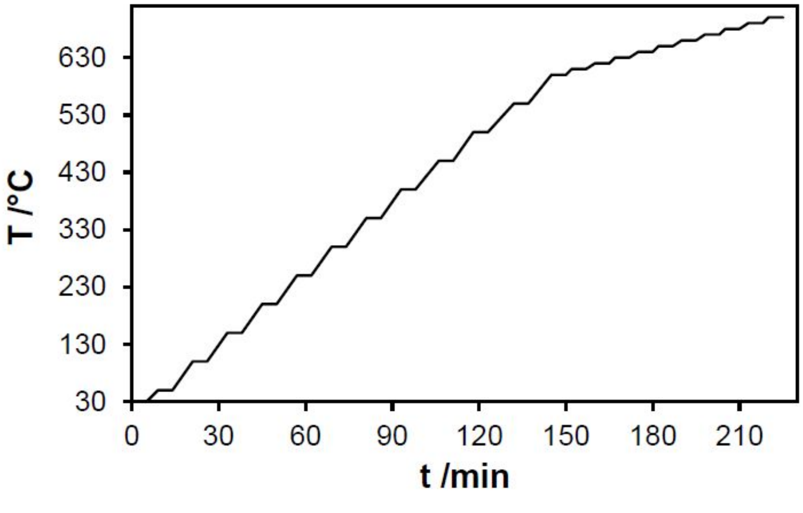

Figure S2 Temperature vs. time curve for the 30 °C- 550 °C *in situ* temperature-dependent experiments. Data were collected for 5 min starting from 30 °C at each 50 °C until 450 °C and at each 50 °C in the 450-550 °C interval where the heating rate was kept constant at 10 °C·min^{-1}.

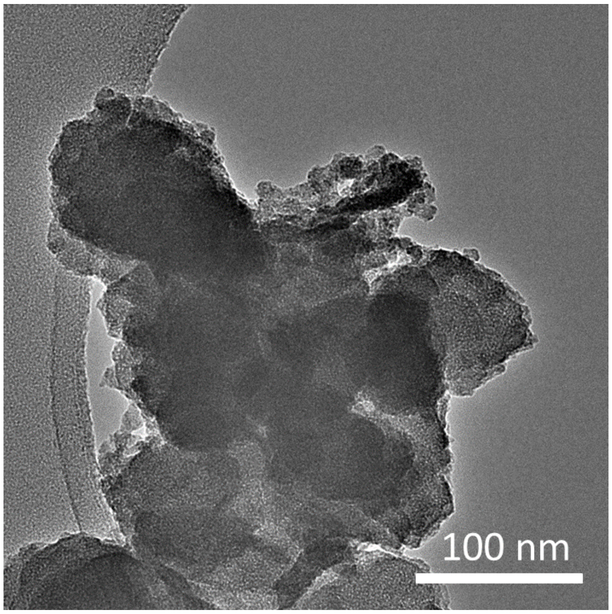

Figure S3 TEM image obtained from *UV- white P* displaying the interconnected porous matrix.

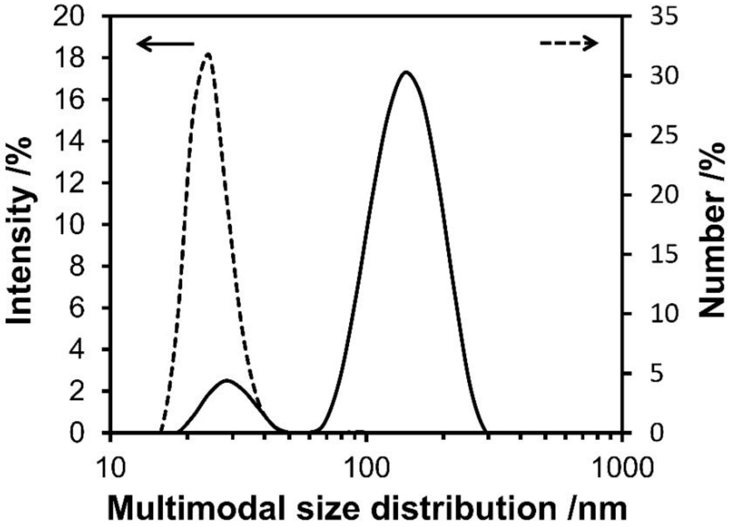

Figure S4 Particle size distribution based on the results of the dynamic light scattering obtained from *UV- white* after ultrasonication. Solid lines show the intensity percentage while dashed lines show the number percentage of the particles. Note that the size values are plotted on a logarithmic scale. The data shows a bimodal particle size distribution centered at 30 nm and at 150 nm. However, the number distribution of the particles indicates that the particle size distribution of *UV-white* is monomodal with 25 nm particle size on average.

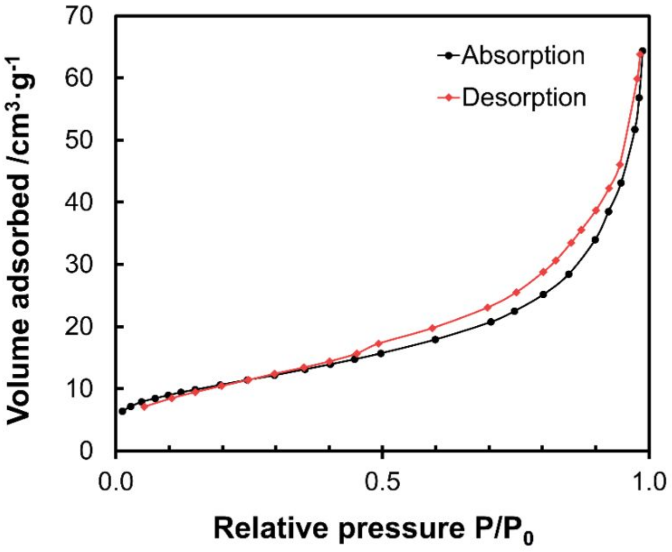

Figure S5 Nitrogen adsorption/desorption isotherms of *UV- white P* sample after degassing approximately 150 mg powder at 150 °C overnight (14 wt% loss).

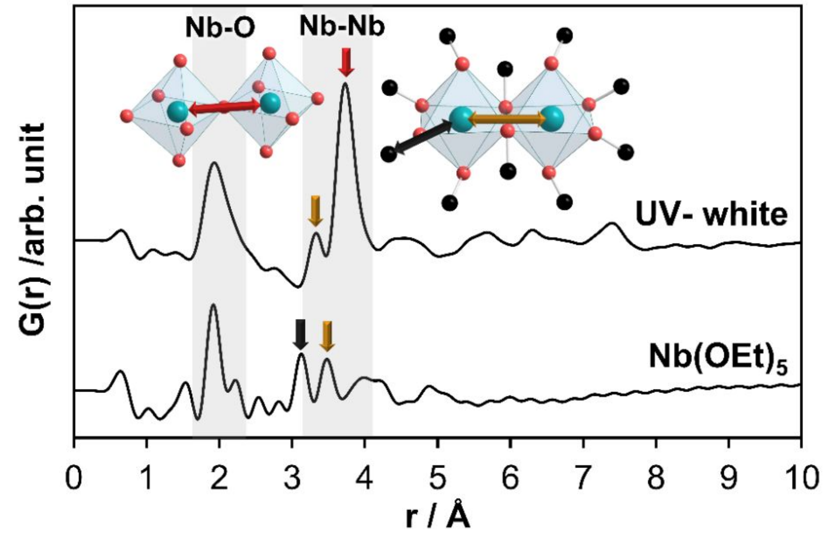

Figure S6 Experimental PDFs obtained from the liquid $Nb(OEt)_5$ precursor measured in a capillary sealed under a protective atmosphere and the *UV- white* sample. The regions containing Nb-O and Nb-Nb distances are marked by grey rectangles. Distances between two Nb atoms of edge- and corner-sharing octahedra are marked by yellow and red arrows. The peak corresponding to Nb-C distances is marked by a black arrow. The disappearance of this pair correlation indicates hydrolysis taking place that most of the Nb-C pair correlations are not present anymore. $Nb_2O_{10}C_{10}$ dimer structure and corner-sharing. The data support the finding of previous studies and confirm the existence of edge-sharing bioctahedral configuration.[1] Note that Nb atoms and O atoms are represented by blue and red spheres. Black spheres represent C atoms.

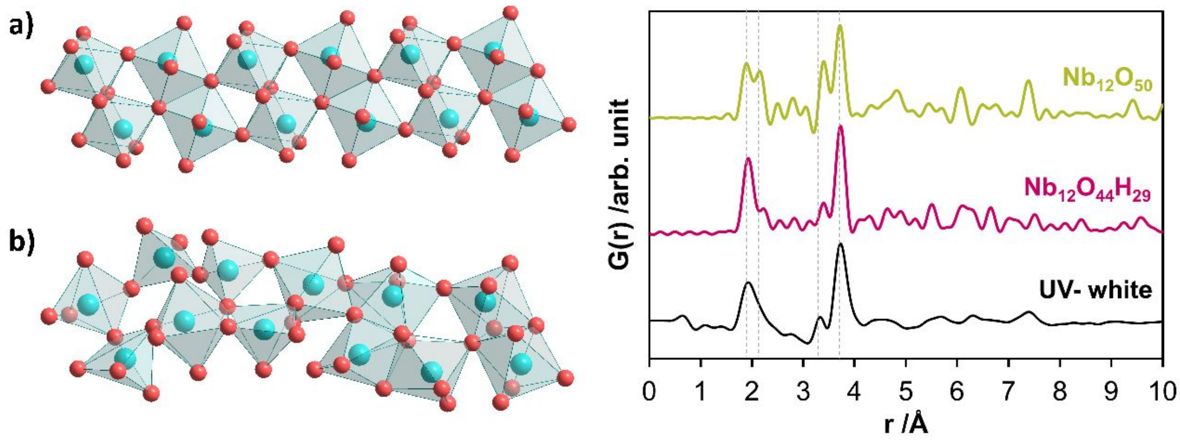

Figure S7 PDFs simulated for (a) $Nb_{12}O_{50}$, a cutout of the B-Nb_2O_5[2] crystal structure and (b) $Nb_{12}O_{44}H_{29}$ cluster model constructed by Llordés *et al.*[3] compared to the experimental PDF obtained from *UV- white*.

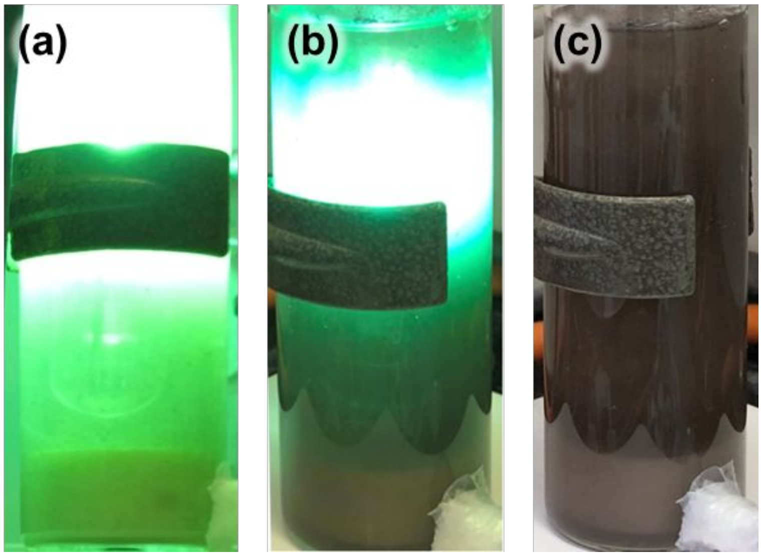

Figure S8 Photographs taken during the preparation of *UV+ dark*. Following the injection of the $Nb(OEt)_5$ precursor into the water-methanol mixture: (a) right after the UV light is turned on, (b) after 5 min and (c) when the UV lamp is turned off after 2 h of UV exposure.

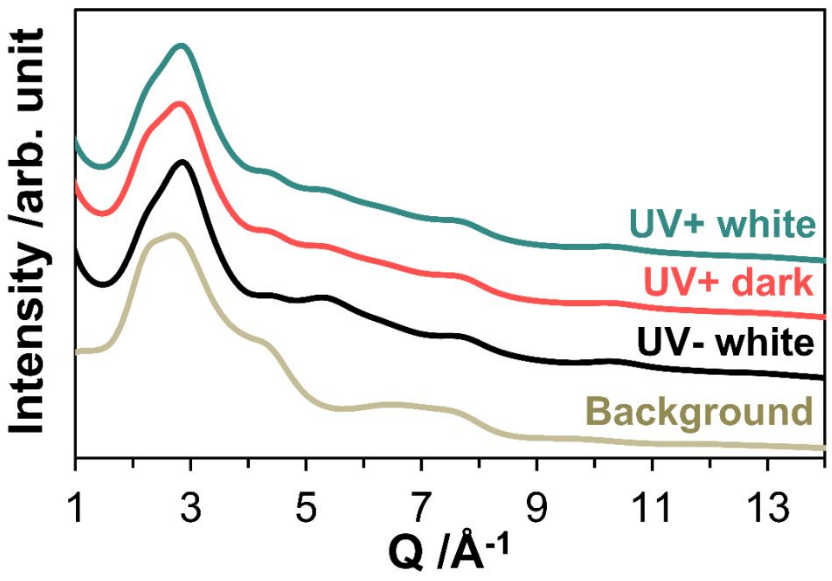

Figure S9 Comparison of the X-ray TS curves obtained from *UV- white* (black curve), *UV+ dark* (coral curve) and *UV+ white* (teal curve) in reaction suspensions in sealed capillaries. Note that the scattering data collected from the empty capillary with the water-methanol mixture is plotted below as a 'background' for comparison. During PDF processing, the contribution from the capillaries was subtracted by excluding Si-O distances associated with the capillary material as the strongest pair correlations.

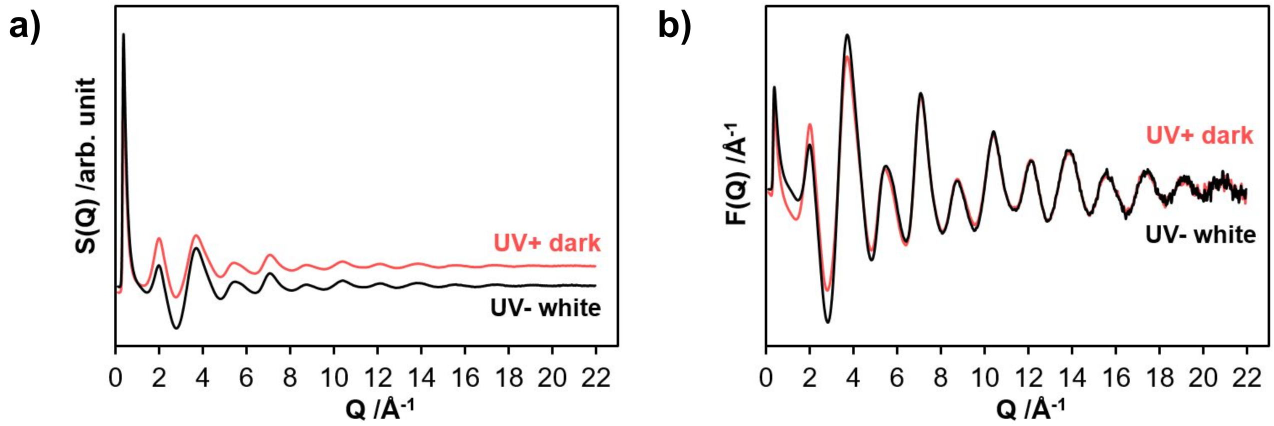

Figure S10 Comparison of the (a) total scattering structure functions (S(Q)) and (b) the reduced structure functions (F(Q)) obtained from *UV- white* (black curve) and *UV+ dark* (coral curve) during processing the total scattering data into PDFs.

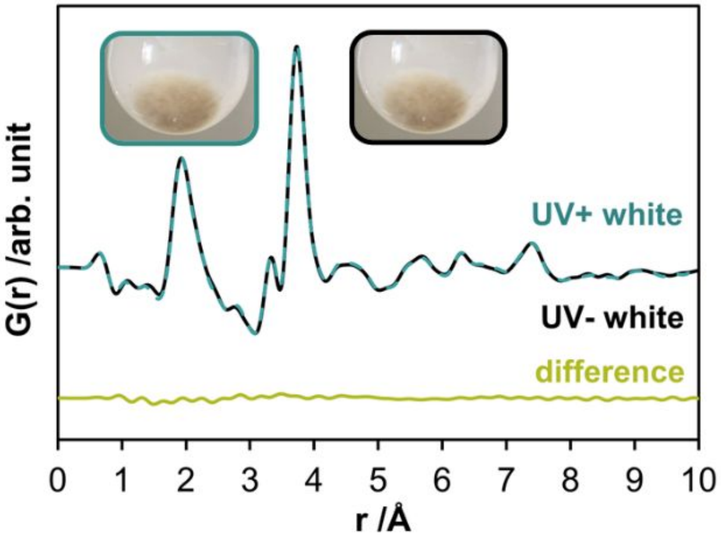

Figure S11 Comparison of the PDFs obtained from *UV- white* (black curve) and *UV+ white* (teal curve) in reaction suspensions in sealed capillaries. The flat difference curve is obtained by subtracting the PDF intensities.

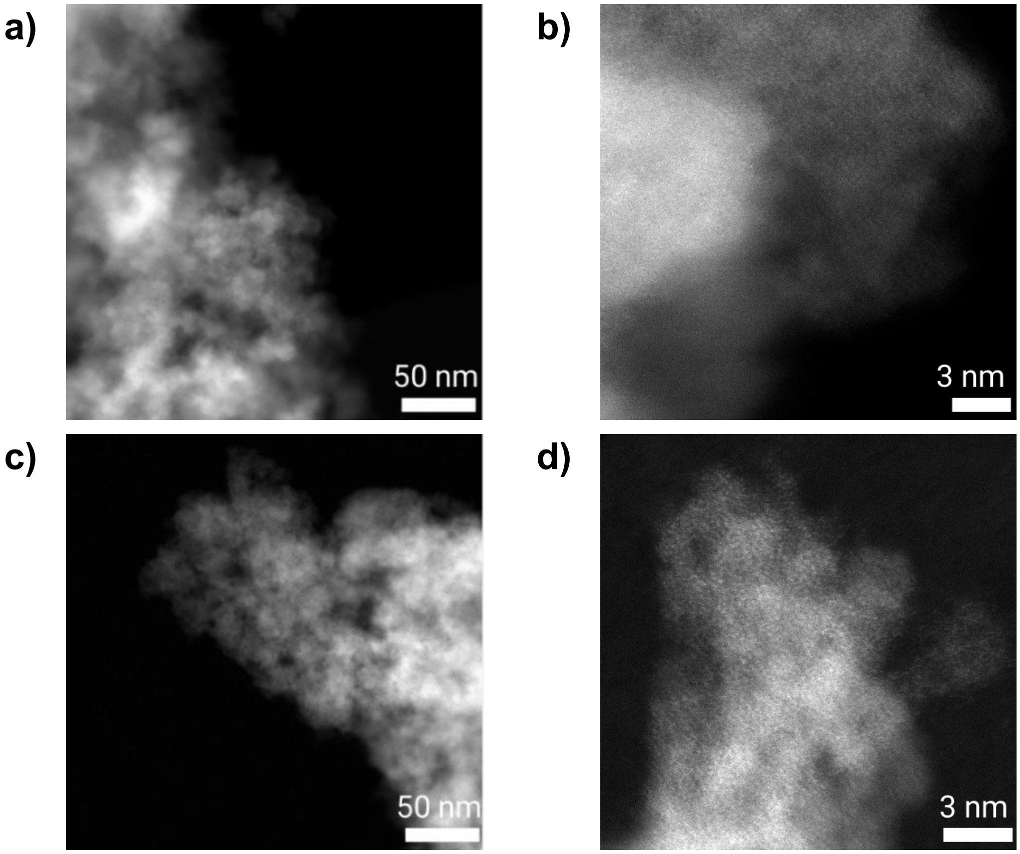

Figure S12 Comparison of morphologies of (a,b) *UV- white P* and (c,d) *UV+ white P* examined by HAADF STEM.

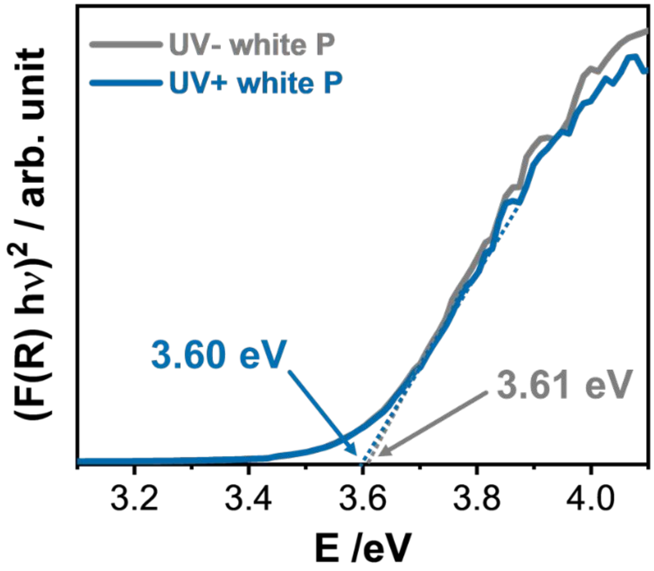

Figure S13 Tauc plots to determine the band gap values of *UV+ white P* and *UV- white P*.

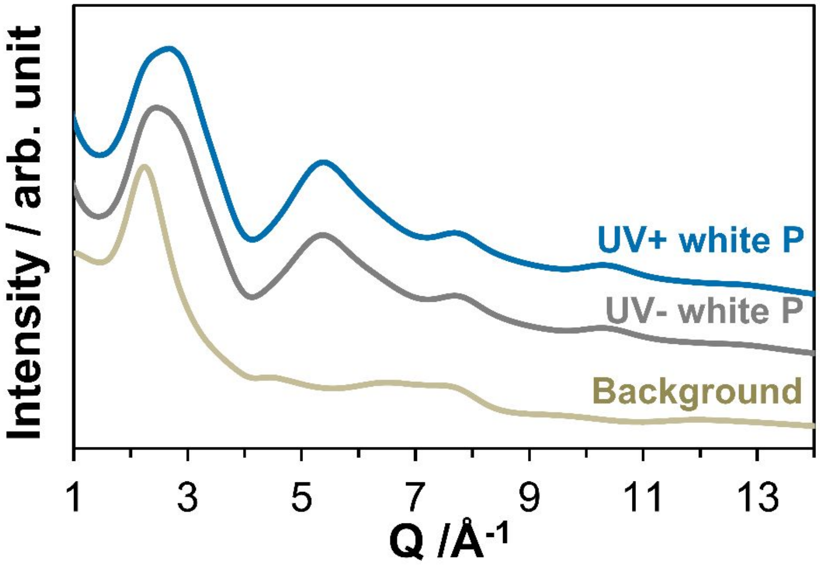

Figure S14 X-ray TS data obtained from *UV+ white P* (blue-colored curve) and *UV- white P* (gray-colored curve) in sealed capillaries. Note that the scattering data collected from the empty capillary is plotted below as 'background' for comparison. During PDF processing, the contribution from the capillaries was subtracted by excluding Si-O distances associated with the capillary material as the strongest pair correlations.

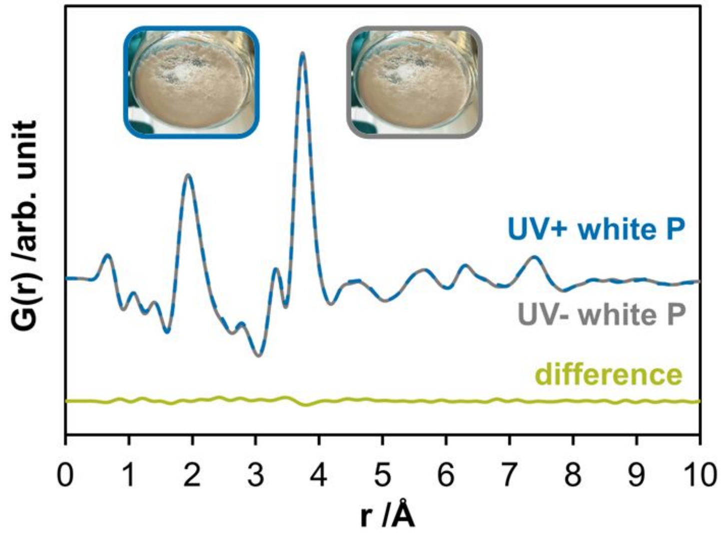

Figure S15 PDFs obtained from *UV+ white P* (blue dashed curve) and *UV- white P* (gray curve) in sealed capillaries. Difference curves are obtained by subtracting the PDF intensities.

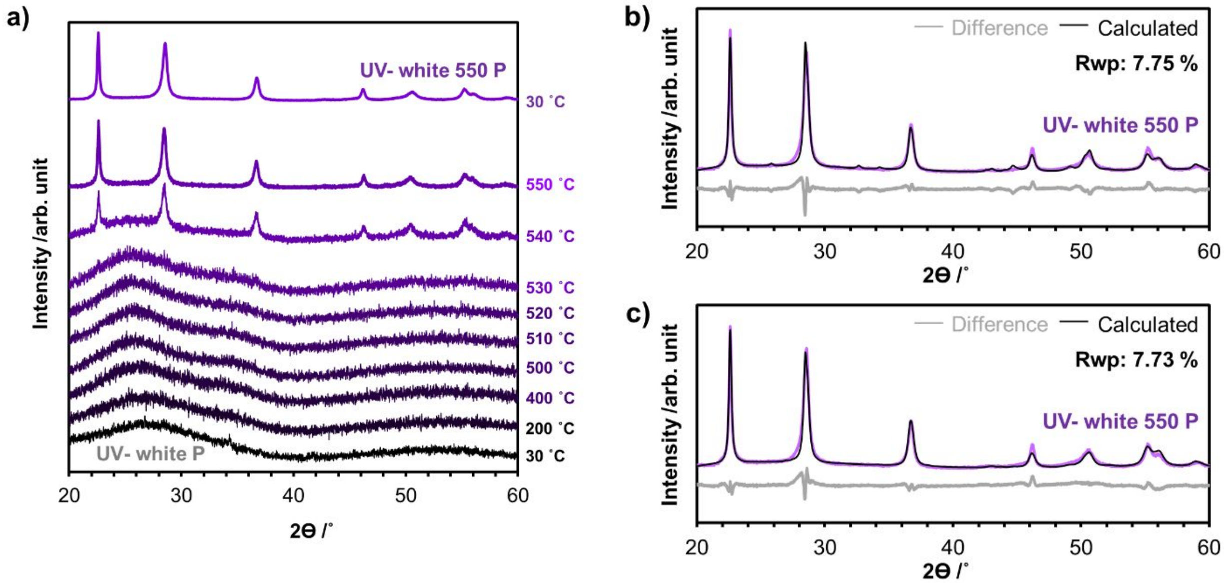

Figure S16 a) Stack of the *in situ* temperature-dependent XRD experiments performed on UV-white P to obtain UV-white 550 P. Data were collected using an in-house instrument (λ= 1.54186 Å). The calculated and difference curves are based on Rietveld refinements performed for UV-white 550 P obtained after cooling to 30 °C, using the crystal structure data of b) T-Nb_2O_5[4] and c) TT-Nb_2O_5.[5]

Table S1 R-values and lattice parameters obtained for UV-white 550 P measured at 30 °C. For the refinement, the crystal structure data of T-Nb_2O_5[4] and TT-Nb_2O_5[5] were used. Data were collected the range 20-60° 2Θ. The simple axial model was applied to model reflection profiles. Thompson-Cox-Hastings pseudo-Voigt was used as the peak shape function. The background was fitted using a 5th-order Chebychev function. Atomic coordinates were not refined and atomic displacement parameters (Beq) were kept constant at 0.5 Å^2.

	T-Nb_2O_5		TT-Nb_2O_5	
**Parameter**	**Initial value**	**Refined value**	**Initial value**	**Refined value**
**R_{wp} /%**	-	7.75	-	7.73
**GOF**	-	3.25	-	3.24
**Lattice parameters /Å**				
**a**	6.175	6.221 (1)	6.209	6.224 (1)
**b**	28.175	29.019 (2)	28.999	29.025 (3)
**c**	3.930	3.932 (1)	3.923	3.932 (1)

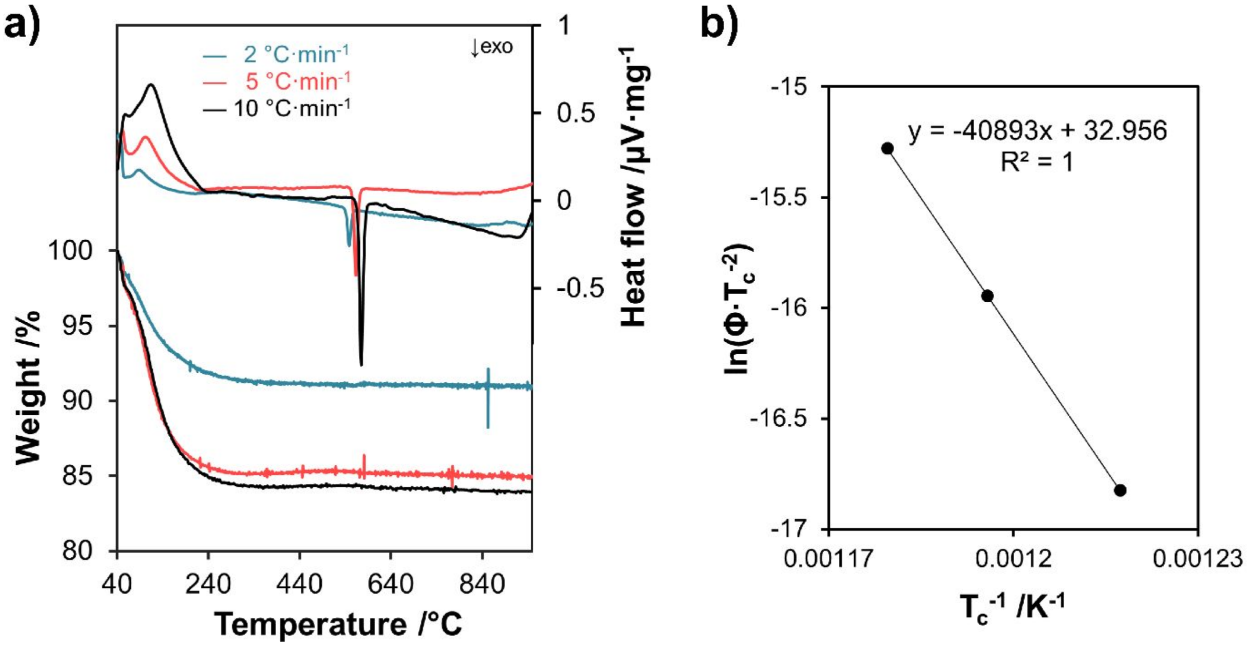

Figure S17 (a) DSC and TG data collected for *UV- white P* under airflow at heating rates of 2 (teal), 5 (coral), and 10 °C·min^{-1} (black). The temperature values corresponding to crystallization peaks are used to construct the (b) Kissinger plot to calculate the activation energy for the crystallization process.[6]

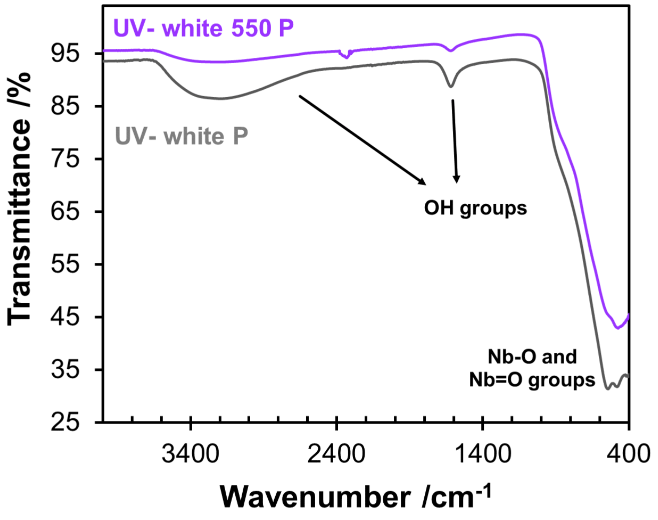

Figure S18 FTIR spectra obtained using ATR mode from *UV- white* 500 P (purple curve) and *UV-white P* (gray curve). Note that both spectra were acquired at room temperature. The comparison points to a decrease in the intensity of bands associated with OH groups (3000-3500 cm^{-1} water-bridged hydroxyl groups, 1630 cm^{-1} H-O-H bending modes). This indicates that the weight loss observed in the TG signal is mainly associated with the removal of water. FTIR spectrum of *UV-white* 500 P displays an additional band around 2300 cm^{-1}, which is attributed to CO_2 groups. Persistent observation of this band might be due to carbonate groups forming at the surface or atmospheric CO_2.

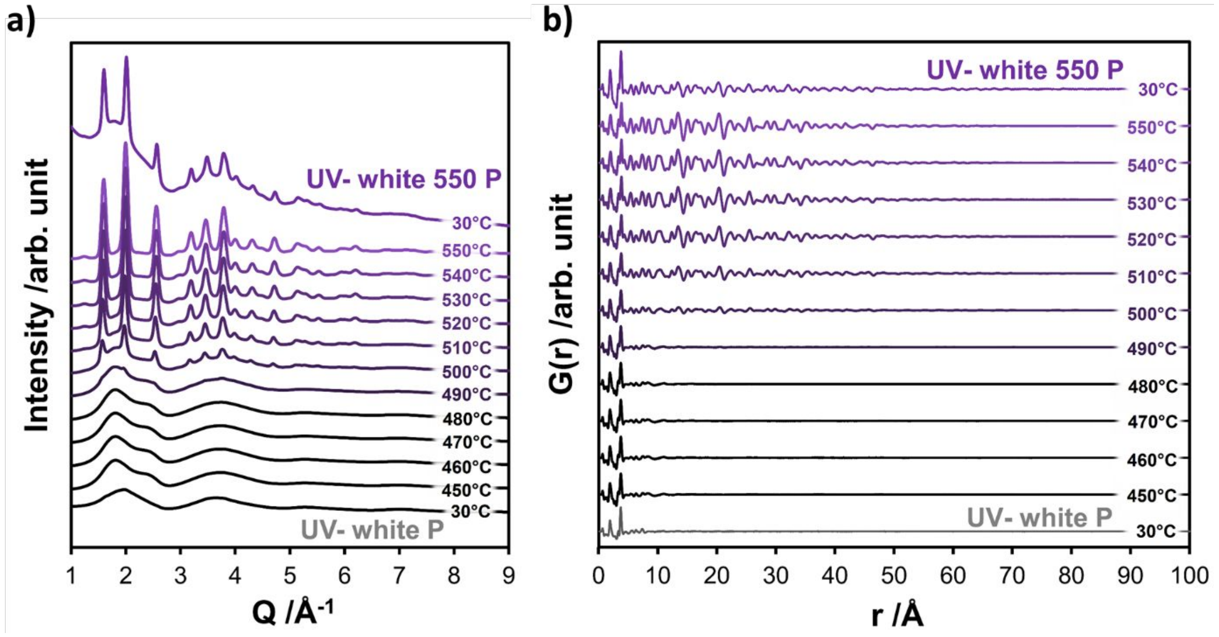

Figure S19 Experimental (a) X-ray total scattering data and (b) the respective long-range PDFs obtained from *in situ* temperature-dependent experiments performed on UV-white P.

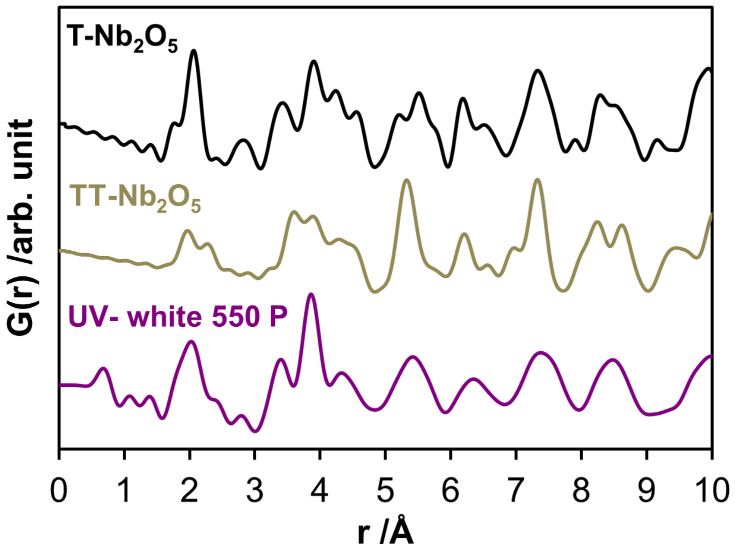

Figure S20 PDFs simulated for T-Nb_2O_5 and TT-Nb_2O_5 crystal structure models presented above the experimental PDF obtained from *UV- white 550 P*.

Table S2 Refined parameters obtained after the final PDF refinement for UV-white 550 P at 30 °C using TT-Nb_2O_5 model structure within the range 8-50 Å (R_w= 0.24). Note that the errors are given in parentheses. The exact parameters were subsequently applied to fit the data further within the range 0.5-20 Å (Rw= 0.79).

**Fit range**		
	TT-Nb_2O_5	
**Parameter**	**Initial value**	**Refined value**
**Quadratic. corr. factor**	1.00	1.01
**Linear corr. factor**	1.00	0.65
**Lattice parameters /Å**		
**a**	6.209	6.198 (0.056)
**b**	28.999	29.569 (0.230)
**c**	3.923	3.914 (0.020)
**Sp-diameter /Å**	80	92 (17)
**Atomic displacement parameters /Å²**		
**Nb**	0.005	0.011 (0.004)
**O**	0.005	0.018 (0.010)

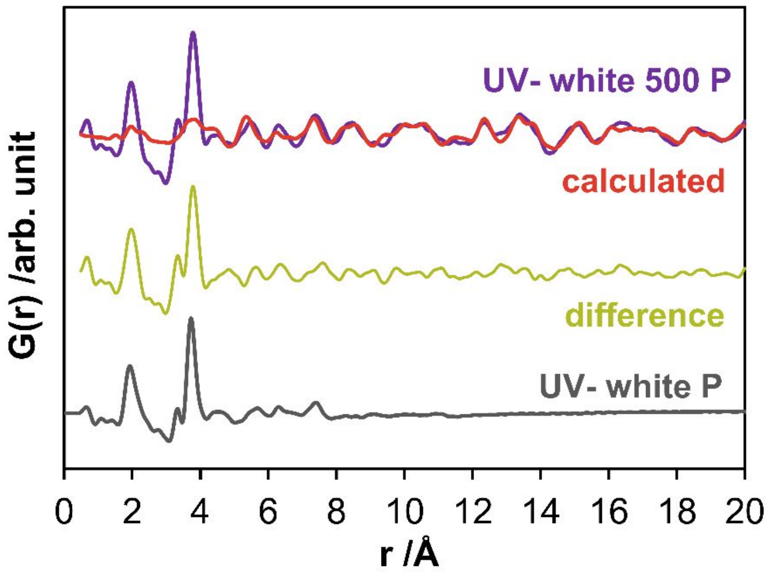

Figure S21 Refinement of the PDFs obtained from *UV- white 550 P* (the sample at 30 °C after heating during the *in situ* temperature-dependent total scattering experiments) using TT-Nb_2O_5 crystalline model structure within the 0.5-20 Å (R_w= 0.79) range when these exact values of the parameters were used in the refinement within the 8-50 Å range were subsequently applied to fit the data in the shorter range. Calculated and difference curves are displayed in red and green. The PDF obtained from *UV- white P* is given below for comparison.

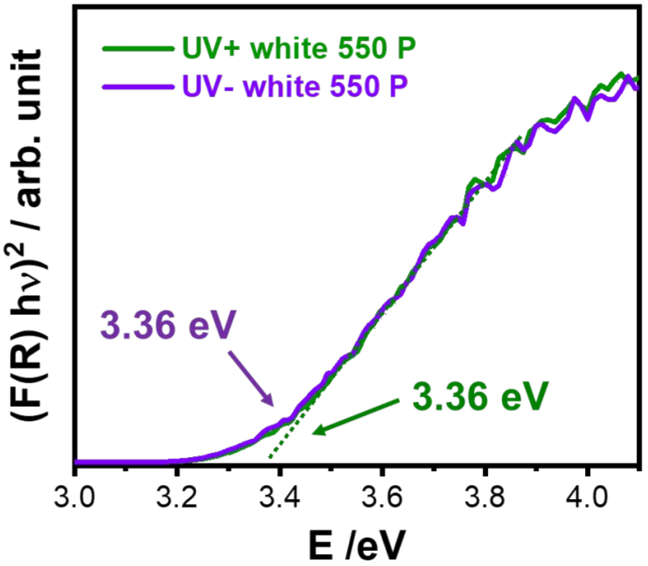

Figure S22 Tauc plots to determine the band gap values of *UV+ white 550 P* and *UV- white 550 P*.

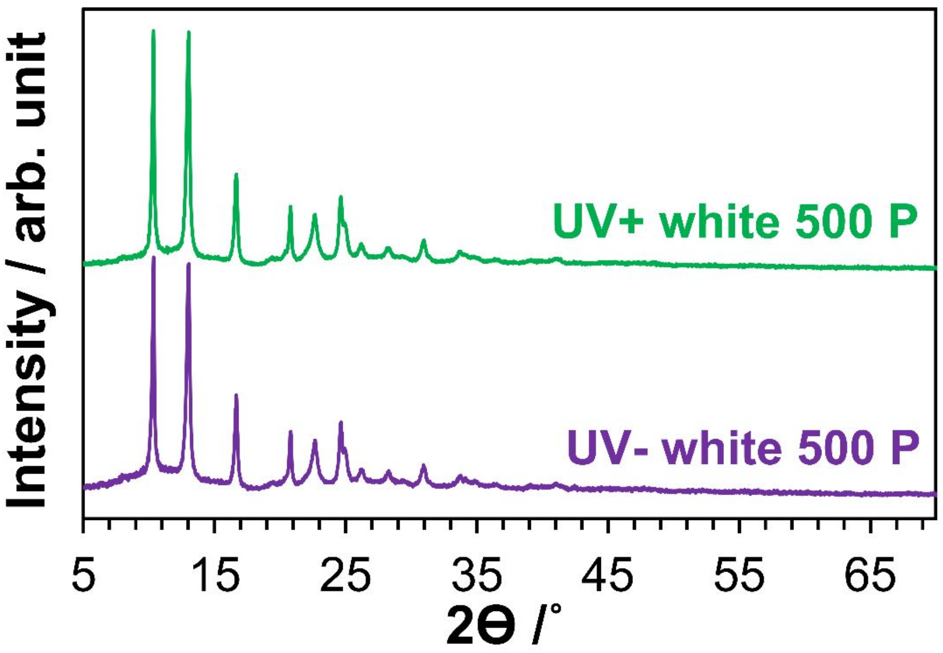

Figure S23 Comparison of the XRD patterns collected from the *UV- white 550 P* and *UV+ white 550 P* using an in-house instrument (λ= 1.541862 Å).

References

(1) Bradley, D. C.; Holloway, C. E. Nuclear magnetic resonance studies on niobium and tantalum penta-alkoxides. *J. Chem. Soc. A* **1968**, 219-223. DOI: 10.1039/J19680000219.

(2) Laves, F.; Petter, W.; Wulf, H. Die Kristallstruktur von ζ-Nb2O5. *Die Naturwissenschaften* **1964**, *51* (24), 633-634. DOI: 10.1007/bf00623669.

(3) Llordés, A.; Wang, Y.; Fernandez-Martinez, A.; Xiao, P.; Lee, T.; Poulain, A.; Zandi, O.; Saez Cabezas, C. A.; Henkelman, G.; Milliron, D. J. Linear topology in amorphous metal oxide electrochromic networks obtained via low-temperature solution processing. *Nat. Mater.* **2016**, *15* (12), 1267-1273. DOI: 10.1038/nmat4734.

(4) Kato, K.; Tamura, S. Die Kristallstruktur von T-Nb_2O_5. *Acta Crystallogr. B* **1975**, *31* (3), 673-677. DOI: 10.1107/S0567740875003603.

(5) Gomes, G. H. M.; Mohallem, N. D. S. Insights into the TT-Nb_2O_5 crystal structure behavior. *Mater. Lett.* **2022**, *318*. DOI: 10.1016/j.matlet.2022.132136.

(6) Kissinger, H. E. Variation of peak temperature with heating rate in differential thermal analysis. *J. Res. Natl. Bur. Stand. (U.S.)* **1956**, *57* (4). DOI: 10.6028/jres.057.026.
